# Redox-switch regulatory mechanism of thiolase from *Clostridium acetobutylicum*

**DOI:** 10.1038/ncomms9410

**Published:** 2015-09-22

**Authors:** Sangwoo Kim, Yu-Sin Jang, Sung-Chul Ha, Jae-Woo Ahn, Eun-Jung Kim, Jae Hong Lim, Changhee Cho, Yong Shin Ryu, Sung Kuk Lee, Sang Yup Lee, Kyung-Jin Kim

**Affiliations:** 1 School of Life Sciences, KNU Creative BioResearch Group, Kyungpook National University, Daegu 702-701, Korea; 2 School of Nano-Bioscience and Chemical Engineering, Ulsan National Institute of Science and Technology (UNIST), Ulsan, 689-798, Korea; 3 Department of Chemical and Biomolecular Engineering (BK21 Plus Program) and BioProcess Engineering Research Center, KAIST, Daejeon 305-701, Korea; 4 Pohang Accelerator Laboratory, Pohang University of Science and Technology, Pohang, Kyungbuk 790-784, Korea; 5 Center for Systems and Synthetic Biotechnology, Institute for the BioCentury, and Bioinformatics Research Center, KAIST, Daejeon 305-701, Korea

## Abstract

Thiolase is the first enzyme catalysing the condensation of two acetyl-coenzyme A (CoA) molecules to form acetoacetyl-CoA in a dedicated pathway towards the biosynthesis of *n*-butanol, an important solvent and biofuel. Here we elucidate the crystal structure of *Clostridium acetobutylicum* thiolase (*Ca*THL) in its reduced/oxidized states. *Ca*THL, unlike those from other aerobic bacteria such as *Escherichia coli* and *Zoogloea ramegera*, is regulated by the redox-switch modulation through reversible disulfide bond formation between two catalytic cysteine residues, Cys88 and Cys378. When *Ca*THL is overexpressed in wild-type *C. acetobutylicum*, butanol production is reduced due to the disturbance of acidogenic to solventogenic shift. The *Ca*THL^V77Q/N153Y/A286K^ mutant, which is not able to form disulfide bonds, exhibits higher activity than wild-type *Ca*THL, and enhances butanol production upon overexpression. On the basis of these results, we suggest that *Ca*THL functions as a key enzyme in the regulation of the main metabolism of *C. acetobutylicum* through a redox-switch regulatory mechanism.

## Abstract (web summary)

*n*-Butanol is a valuable biofuel that can be produced industrially by bacterial fermentation. Here the authors uncover a redox-switch within *Clostridium acetobutylicum*'s thiolase—a key enzyme involved in *n*-butanol biosynthesis—that controls the rate of fermentative butanol production.

## Graphical abstract

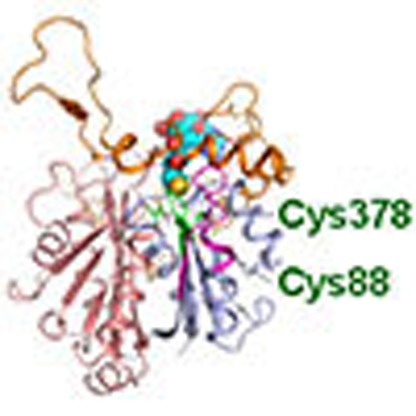

Fermentative *n-*butanol production was first reported by Louis Pasteur in 1861, and the bioprocess was industrialized using *Clostridium acetobutylicum* isolated by Chaim Weizmann in the early 1900s (ref. 1). The ‘acetone–butanol–ethanol' (ABE) fermentation by *Clostridium* strains has been known to be the most efficient process for *n-*butanol production^2^,^3^. Due to the growing worldwide issues such as energy security and climate change, the biotechnological production of *n-*butanol has been receiving much renewed interest. This is because *n*-butanol possesses much better fuel characteristics compared with ethanol, such as higher energy content, less corrosiveness, less hygroscopy and easier blendability with gasoline and diesel fuels^4^,^5^,^6^.

It has been known that in anaerobic bacteria of the genus *Clostridium n*-butanol is synthesized from acetyl-coenzyme A (CoA) via six tightly regulated steps that are catalysed by independent proteins^7^. For more than two decades, numerous engineering attempts were made, ranging from genetic modifications to process optimization, aimed at improving *n*-butanol production by *Clostridium* strains. Despite all such effort, *n-*butanol titres obtained were <20 g l^−1^ (ref. 8), which needs to be increased for competitive commercialization. To understand the molecular mechanisms that regulate the *n*-butanol biosynthetic pathway and to enhance the yield of *n*-butanol, enzymes in the *n*-butanol biosynthetic pathway have been introduced into commonly used industrial hosts such as *Escherichia coli*, *Pseudomonas putida* and *Bacillus subtilis*, because their genetic and physiological characteristics are well defined and various genetic tools exist to favour their modifications^9^,^10^,^11^.

To enhance *n*-butanol production by *Clostridium* strains or engineered heterologous host strains alike, detailed understanding of the reaction and regulatory mechanisms of key enzymes involved in the *n*-butanol biosynthetic pathway is necessary^12^,^13^. However, our understanding on the regulatory mechanisms of the enzymes in the *n-*butanol biosynthetic pathway in *Clostridium* is rather limited. Here, we present the crystal structure of *C. acetobutylicum* thiolase (*Ca*THL), a type II biosynthetic thiolase that catalyses the first step of *n-*butanol biosynthesis and condenses two acetyl-CoAs to acetoacetyl-CoA. We reveal that *Ca*THL, unlike other type II biosynthetic thiolases from aerobic bacteria, is regulated via modulation of a redox-switch, indicating that *n-*butanol production and the relevant metabolic fluxes in *Clostridium* are tightly regulated by thiolase.

## Results

### Structure of the oxidized form of *Ca*THL

**Figure 1:**
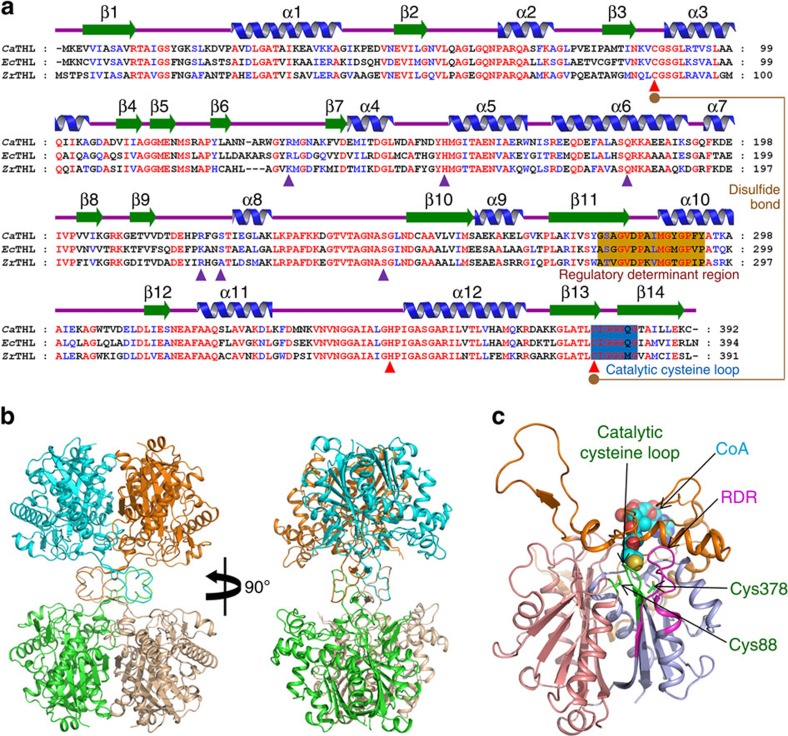
Crystal structure of *Ca*THL. (**a**) Amino acid sequence alignment of thiolases. Secondary structure elements are drawn on the basis of reduced form of *Ca*THL structure and shown with a green-coloured arrow (β-sheet) and blue-coloured helix (α-helix). The ‘regulatory determinant region (RDR)' and the ‘catalytic cysteine loop' are shown in orange and blue colour boxes, respectively. Residues involved in enzyme catalysis and CoA binding are indicated by red- and blue-coloured triangles, respectively. Two catalytic cysteine residues, Cys88 and Cys378, involved in a disulfide bond formation are shown as orange-coloured circles, and the disulfide bond is indicated as an orange line. *Ca*THL, *Ec*THL and *Zr*THL are representations of THL from *C. acetobutylicum*, *E. coli* and *Z. ramigera*, respectively. (**b**) Tetrameric structure of *Ca*THL. The tetrameric structure is shown as a ribbon diagram showing one dimmer in blue and orange and the other dimmer in green and salmon. Two tightly interacted dimmers form a tetramer through L-domain. (**c**) Monomeric structure of *Ca*THL. A monomeric protein is shown as a ribbon representation in which the N-, C- and L-domains are distinguished with salmon, light blue and orange colours, respectively. The ‘catalytic cysteine loop' and ‘RDR' are distinguished with green and magenta colours, respectively, and labelled. The bound-CoA molecule is shown as sphere model, and two catalytic cysteine residues are presented as stick models with green colour.

The 1.77 Å crystal structure of *Ca*THL was determined to understand the structural basis for its catalytic and regulatory mechanisms. The asymmetric unit contains two *Ca*THL molecules, and the tetrameric structure of the protein could easily be generated by applying crystallographic *P*2_1_2_1_2 symmetry (Fig. 1 and Table 1). The overall monomeric structure of *Ca*THL shares the general fold architecture found in the type II biosynthetic thiolase family of proteins^14^,^15^. *Ca*THL consists of three domains: an N-terminal α/β domain (N-domain, residues 1–119 and 249–269), a loop domain (L-domain, residues 120–248), and a C-terminal α/β domain (C-domain, residues 270–392). The N- and C-domains form a typical five-layered fold (α–β–α–β–α) observed in other thiolase superfamily of proteins including peroxisomal degradative thiolase from *Saccharomyces cerevisiae*, β-ketoacyl synthase II from *E. coli*, and the biosynthetic thiolase from *Zoogloea ramigera* (*Zr*THL)^14^,^15^,^16^,^17^,^18^,^19^,^20^. The L-domain displays an α/β fold and is associated mainly with the C-domain (Fig. 1c).

**Figure 2:**
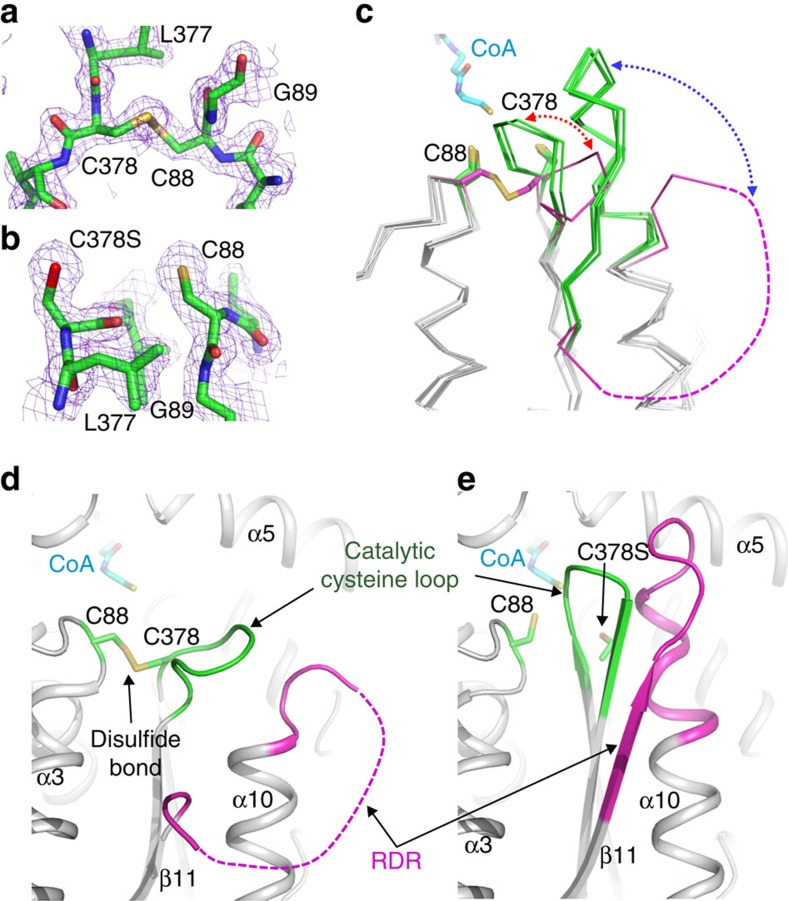
Structural features of the redox-switch modulation of *Ca*THL. (**a**,**b**) Reversible disulfide bond formation. Disulfide bond formation in the oxidized form (**a**), and a breakage of the disulfide bond in the reduced form (**b**) of *Ca*THL are shown in a stick model, and their omit electron densities (purple mesh) are contoured at 2.0*σ*. (**c**) Structural changes of the ‘catalytic cysteine loop' and the ‘regulatory determinant region (RDR)'. The ‘catalytic cysteine loop' and the RDR of the oxidized and the reduced forms of *Ca*THL, *Ec*THL, *Zr*THL (pdb code 1DLV) and the *Ca*THL^V77Q/N153Y/A286K^ mutant are superimposed, and shown as ribbon models. The ‘catalytic cysteine loop' and the ‘regulatory determinant region (RDR)' of the oxidized form of *Ca*THL are shown with magenta colour, and those of other thiolases are with a green colour. The disordered RDR of the oxidized form of *Ca*THL is shown as a dotted line with magenta colour. The disulfide bond in the oxidized form and the two cysteine residues in the reduced form of *Ca*THL are shown in a stick model and labelled appropriately. The bound-CoA molecule is presented as a stick model with cyan colour. The structural changes of the ‘catalytic cysteine loop' and the RDR of *Ca*THL are shown as red and blue dotted-arrow lines, respectively. (**d**,**e**) Structural changes of *Ca*THL. The ‘catalytic cysteine loop' and the RDR of the oxidized (**d**) and reduced (**e**) form of *Ca*THL are shown as a ribbon diagram. The ‘catalytic cysteine loop' and the RDR of *Ca*THL are distinguished with green and magenta colours, respectively, and labelled. The two catalytic cysteine residues and the CoA molecule are shown as stick models with green and cyan colours, respectively. The secondary structure elements are labelled appropriately.

Even though *Ca*THL and *Zr*THL showed similar overall fold architectures, a remarkable difference was observed at the active site of *Ca*THL. Interestingly, under oxidized conditions, a disulfide bond was formed between two catalytic cysteine residues (Cys88 and Cys378) in *Ca*THL (Fig. 2a,c,d, and Supplementary Fig. 1). Such disulfide bond formation has not been reported in other type II biosynthetic thiolases. The disulfide bond formation was followed by a large conformational change in the catalytic cysteine loop (CIGGGQG, residues 378–384). The Cys378 residue rotates 180° towards the Cys88 residue via the rotation of the peptide bond between Leu377 and Cys378, which in turn induces a movement of the catalytic cysteine loop away from the active site. The consequence of this conformational change in the catalytic cysteine loop is that the region between β11 and α10 (SAGVDPAIMGYGP, residues 280–292) becomes disordered due to the steric clash (Fig. 2c,d). Hereafter, this disordered region will be referred to as the flexible region. In summary, the oxidized *Ca*THL becomes inactive due to the oxidation of both catalytic cysteine residues accompanied by large conformational changes in the catalytic cysteine loop and the flexible region.

### Structure of the reduced form of *Ca*THL

The unusual disulfide bond formation and large structural changes observed in the oxidized form of *Ca*THL (but not in *Zr*THL) raised the possibility of the influence of the redox state on the folding kinetics of *Ca*THL. To further investigate the role of the redox state on the structural properties of *Ca*THL, the crystal structure of the reduced form of *Ca*THL was also determined using the C378S mutant *Ca*THL, a protein that cannot form a disulfide bond. The active state configuration of the reduced form of *Ca*THL was similar to that of *Zr*THL, in which the two cysteine residues involved in the disulfide bond formation in the oxidized form positioned apart with a distance of ∼6.4 Å (Fig. 2b,c,e). The catalytic cysteine loop in the reduced form of *Ca*THL moved back to the active site by ∼8 Å. Accordingly, the flexible region was found to be highly ordered, forming the native sheet–turn–helix conformation as observed in *Zr*THL (Fig. 2c,e). In the reduced form of *Ca*THL, the positions of two catalytic cysteine residues and the conformation of the catalytic cysteine loop were restored, resulting in an active form of the enzyme.

Next, the crystal structure of the reduced form of *Ca*THL in complex with CoA was determined. The catalytic residues such as Cys88, Cys378 and His348 were observed to be located at the same positions as those of the corresponding residues of *Zr*THL, indicating that the catalytic mechanism might be identical to that of *Zr*THL. However, the CoA-binding mode of *Ca*THL was quite unique when compared with that of *Zr*THL. In *Ca*THL, His218 residue, whose corresponding residue is Tyr218 in *Zr*THL, is involved in the stabilization of pyrophosphate moiety of CoA, and Ser223 residue is located at the position of Ala223 in *Zr*THL. Moreover, in *Ca*THL, Arg133 residue from the neighbouring polypeptide is involved in the stabilization of pyrophosphate moiety of CoA through hydrogen bond, whereas the corresponding Lys133 residue in *Zr*THL has conformation distal from the bound CoA (Supplementary Fig. 2).

### Redox-switch regulatory mechanism of *Ca*THL

**Figure 3:**
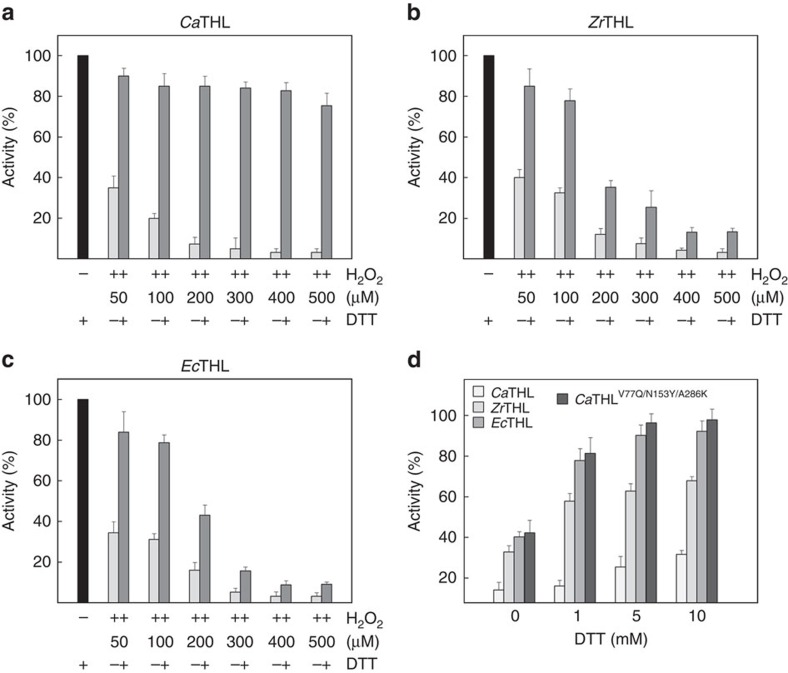
*Ca*THL senses redox environment through a reversible disulfide bond. (**a**–**c**) Activity recovery test of *Ca*THL, *Zr*THL and *Ec*THL proteins. To investigate how thiolases from different organisms sense redox environment, *Ca*THL, *Zr*THL and *Ec*THL proteins were treated with various concentrations of H_2_O_2_ and thiolase activity was measured. DTT with 10 mM final concentration was then added to switch the environment to a reduced state, and compared the activity recovery. Over 80% of activity was recovered from *Ca*THL that treated with even 500 μM H_2_O_2_ (**a**), whereas almost no activity was recovered from *Zr*THL and *Ec*THL that treated with more than 400 μM H_2_O_2_ (**b**,**c**). (**d**) Activity of four thiolases at various concentrations of DTT. Thiolase activity of *Ca*THL proteins of wild-type and the *Ca*THL^V77Q/N153Y/A286K^ mutant, *Zr*THL and *Ec*THL were measured with various concentrations of DTT (0, 1, 5 and 10 mM). While the wild-type *Ca*THL protein shows only ∼30% activity compared with those of *Zr*THL and *Ec*THL, the activity of the *Ca*THL^V77Q/N153Y/A286K^ mutant shows more than threefold increase compared with the wile-type enzyme, which were correspondent to similar or even higher activity compared with *Ec*THL and *Zr*THL. Error bars indicate standard deviation among three independently repeated experiments.

In general, redox-mediated modification of cellular proteins confers a response to reactive oxygen species, and the changes in response to redox status regulate the initiation of signal transduction pathways and the induction of gene expression^21^,^22^,^23^. The crystal structures of oxidized and reduced forms of *Ca*THL and its complex with CoA suggest that, unlike other known type II biosynthetic thiolases, *Ca*THL is regulated by a redox-switch modulation through reversible disulfide bond formation. To verify a redox-switch modulation and the disulfide bond formation in *Ca*THL, the susceptibility of different thiolases, including *Ca*THL, *Zr*THL and *Ec*THL, to hydrogen peroxide (H_2_O_2_), which causes the loss of enzyme activity, was examined. When the thiolases were treated with various concentrations of H_2_O_2_, all three of them lost the activity in the presence of H_2_O_2_ at 100 μM or higher concentration. When dithiothreitol (DTT) (10 mM) was added to switch the protein to a reduced state, the activity of *Ca*THL was almost completely recovered for all concentrations of H_2_O_2_, whereas the activities of *Zr*THL and *Ec*THL could not be completely recovered when H_2_O_2_ concentration was higher than 100 μM (Fig. 3a–c). These results indicate that *Ca*THL is reversibly inactivated by oxidative stress through the disulfide bond formation between the two catalytic residues, Cys88 and Cys378, which enables the enzyme to protect its active site from oxygen radicals and be reactivated upon switching back to a reducing condition. The redox titration experiments were performed to determine the redox potential of the disulfide in CaTHL, and was −269.54 mV (Supplementary Fig. 3). On the other hand, *Zr*THL and *Ec*THL seem to be permanently inactivated upon the oxidation of the corresponding catalytic cysteine residues to sulfinic or sulfonic acid, and cannot be switched back to the active enzymes under reducing condition.

**Figure 4:**
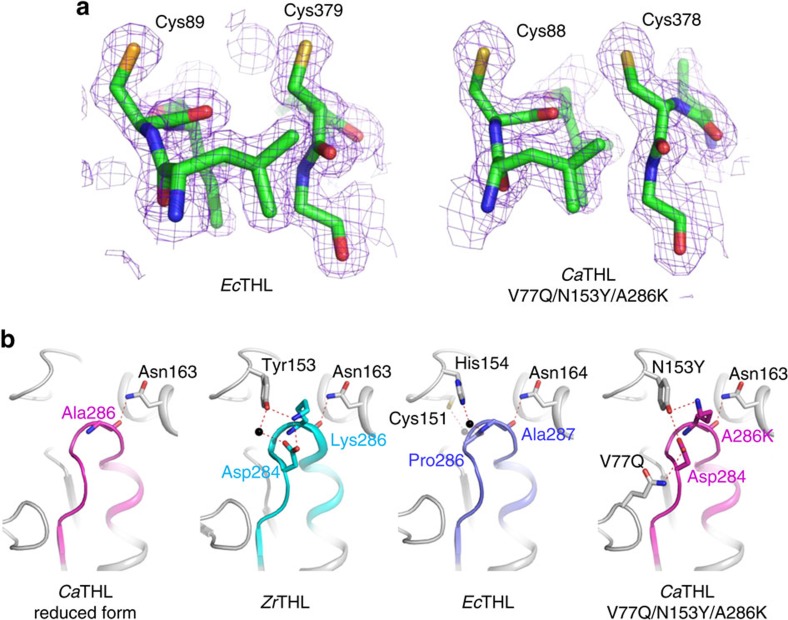
Stabilization of the regulatory determinant region (RDR) of thiolases. (**a**) Electron density map of the region containing two catalytic cysteine residues in *Ec*THL and the *Ca*THL^V77Q/N153Y/A286K^ mutant. Two catalytic cysteine residues of *Ec*THL and the *Ca*THL^V77Q/N153Y/A286K^ mutant are shown as stick models, and their omit electron densities (purple mesh) are contoured at 2.0*σ*. In *Ec*THL and the *Ca*THL^V77Q/N153Y/A286K^ mutant, the disulfide bond formation was not observed even under an oxidized condition. (**b**) Hydrogen bonding of RDRs with surrounding regions. RDRs and their surrounding regions are shown as ribbon diagrams. The RDRs of *Ca*THL and those of *Zr*THL (PDB code 1DLV) and *Ec*THL are shown as magenta, cyan and light blue colours, respectively. Four thiolases (reduced form of *Ca*THL, *Zr*THL, *Ec*THL and *Ca*THL^V77Q/N153Y/A286K^) are labelled appropriately. Residues involved in the stabilization of RDRs are shown as a stick model, and labelled. Hydrogen bonds are presented as red-coloured dotted lines.

Structural studies on *Zr*THL have shown that the catalytic Cys89 residue, corresponding to the Cys88 residue of *Ca*THL, oxidizes to sulfenic acid rather than forming a disulfide bond under oxidized condition^19^, which is responsible for the permanent loss of its activity. Thus, *Zr*THL is a non-redox-switch regulated enzyme. For comparison, the crystal structure of *Ec*THL was also determined at 2.10 Å resolution (Supplementary Fig. 4). As hypothesized, there was no disulfide bond formation observed between the two catalytic cysteine residues, Cys89 and Cys379, under oxidized condition (Fig. 4a). In addition to the absence of a disulfide bond, the catalytic cysteine loop and the flexible region had similar conformations to those of the reduced form of *Ca*THL, confirming that *Ec*THL is also non-redox-switch regulated (Fig. 2c). Taken together, unlike *Zr*THL and *Ec*THL, *Ca*THL senses the oxidative stress and protects its active site via reversible disulfide bond formation; this redox-switch modulation is a unique feature of *Ca*THL identified for the first time.

### Regulatory determinant region of thiolases

Having found the redox-switch regulatory mechanism of *Ca*THL, it was aimed to elucidate the structural motif responsible for conferring such unique mechanism, as compared with other type II biosynthetic thiolases that possess the same catalytic mechanisms, similar overall domain architectures, and highly similar amino acid sequence identities. It was hypothesized that a detailed structural comparison of the flexible regions of *Ca*THL, *Ec*THL and *Zr*THL might provide an answer to this question. The flexible regions of *Ec*THL and *Zr*THL are well stabilized by direct and water-mediated hydrogen bond network; on the other hand, in *Ca*THL, strong hydrogen bond interactions are not observed between the flexible region and its surrounding region. Absence of hydrogen bonds might cause less stability of the flexible region in *Ca*THL (Fig. 4b).

Considering that the flexible region of *Ca*THL undergoes an order-to-disorder conformational change depending on the disulfide bond formation, it seems that the stability of the flexible region of *Ca*THL is not sufficiently high, leading to disulfide bond formation under oxidized condition. This would confer the redox-switch regulatory mechanism to the enzyme; on the other hand, the highly stabilized flexible regions of *Ec*THL and *Zr*THL disable the rotation of the Cys378 residue and hence prevent disulfide bond formation. Taken together, it can be concluded that the stability of the flexible region determines the regulatory mechanism of THL, and thus, the flexible region can be considered as a regulatory determinant region (RDR).

### Role of *Ca*THL during the phase transition

It is well known that the fermentation of *C. acetobutylicum* shows two distinct phases: acidogenic phase where acetic and butyric acids are produced, followed by solventogenic phase where the acids produced are reassimilated and converted to *n*-butanol and ethanol with concomitant production of acetone. *Ca*THL competes with phosphotransacetylase involved in acetic acid biosynthesis for acetyl-CoA to produce butyric acid during acidogenic phase, while competes with aldehyde alcohol dehydrogenase during solventogenic phase. Thus, regulation of thiolase activity in *C. acetobutylicum* can contribute to controlling the ATP yield and NAD^+^ regeneration at the acetyl-CoA node (Supplementary Fig. 5). Intracellular *Ca*THL can be regulated by CoASH, butyryl-CoA and ATP; *Ca*THL is sensitive to CoASH at micro-molar level, and to butyryl-CoA and ATP at 1 and 10 mM, respectively^24^. It has been reported that solventogenesis is induced by a certain intracellular level of butyric acid and butyryl phosphate^25^,^26^,^27^ as well as the change of culture pH (refs 28, 29, 30). These previous findings might be explained by the newly discovered redox-switch regulatory mechanism of *Ca*THL, which might be involved in the regulation of switching from acidogenic phase to solventogenic phase in *C. acetobutylicum*.

**Figure 5:**
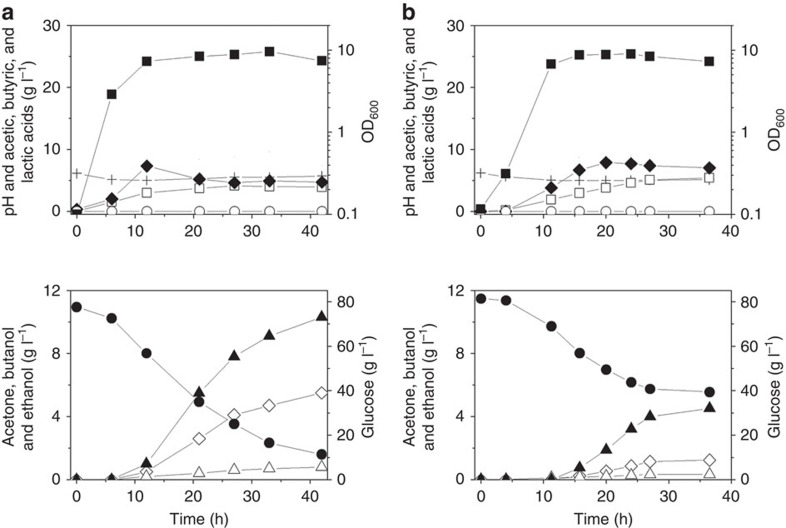
*In vivo* characterization of the redox-switch regulatory mechanism of the *Ca*THL. The *thl*_*ca*_ gene was overexpressed in the biphasic *C. acetobutylicum* type strain (ATCC 824), to see if the overexpression of *Ca*THL affects the metabolism with respect to metabolic flux distribution and the acidogenic to solventogenic phase transition. Anaerobic batch fermentation profiles of the biphasic *C. acetobutylicum* ATCC 824 (**a**) and the ATCC 824 strain overexpressing the *Ca*THL (**b**) are shown. Symbols are: acetic acid (open squares), butyric acid (filled diamonds), lactic acid (open circles), pH (crosses), OD_600_ (filled squares), glucose (filled circles), acetone (open diamonds), ethanol (open triangles) and *n*-butanol (closed triangles). Fermentations were performed at least in duplicates showing reproducibility, and the profiles shown are that of one representative fermentation.

On the basis of this hypothesis, the effects of *Ca*THL overexpression on the fermentation profile were examined. When the *thl*_*Ca*_ gene was overexpressed in the *C. acetobutylicum* wild-type strain ATCC 824 under the control of its native promoter, *n*-butanol production (4.5 g l^−1^) was decreased compared to the wild-type strain (10.7 g l^−1^) (Fig. 5). On the other hand, butyric acid (7.1 g l^−1^) and acetic acid (5.4 g l^−1^) concentrations were higher than those (4.4 and 3.7 g l^−1^, respectively) obtained with the wild-type strain (Fig. 5). Butyric and acetic acids were hardly reassimilated (Fig. 5) with the low acetate uptake flux (*rACUP*) and butyrate uptake flux (*rBUUP*) (Supplementary Figs 5 and 6), indicating that overexpression of *Ca*THL disturbs proper phase transition from acidogenesis to solventogenesis. *C. acetobutylicum* overexpressing *Ca*THL cannot reassimilate butyrate promptly, which causes the genes responsible for solventogenic enzymes uninduced to respond to butyrate production. For example, the expression of CoA transferase involving acid reassimilation was not detected during phase transition from acidogenic to solventogenic phases by two-dimensional gel electrophoresis (Supplementary Figs 7 and 8).

### Structure-based change of *Ca*THL

To further examine the redox-switch regulatory mechanism of *Ca*THL and its effect on the regulation of *C. acetobutylicum* physiology, a non-redox-regulated *Ca*THL mutant was generated. On the basis of the finding that the instability of RDR confers the redox-switch regulatory mechanism to *Ca*THL, a *Ca*THL mutant *Ca*THL^V77Q/N153Y/A286K^ was generated by introducing residues that might be able to form hydrogen bonds with their neighbours and subsequently increase the stability of the *Ca*THL RDR. Then, the activity recovery test was performed for the *Ca*THL^V77Q/N153Y/A286K^ mutant. Unlike the wild-type *Ca*THL, the activity of the H_2_O_2_-treated *Ca*THL^V77Q/N153Y/A286K^ mutant could not be recovered by the addition of a reducing agent as similarly found for *Ec*THL and *Zr*THL (Supplementary Fig. 9).

To confirm the reason for this, the crystal structure of the *Ca*THL^V77Q/N153Y/A286K^ mutant was determined at 2.3 Å resolution; it was structurally confirmed that there were the change of regulatory mechanism and the stability of RDR in the *Ca*THL^V77Q/N153Y/A286K^ mutant. As expected, a disulfide bond formation was not observed between the catalytic cysteine residues in the *Ca*THL^V77Q/N153Y/A286K^ mutant and the RDR was found to be tightly ordered, forming a sheet–turn–helix conformation even without the addition of a reducing agent (Fig. 4a). More interestingly, the mutated residues, V77Q, N153Y and A286K, were positioned to form hydrogen bonds with each other (Fig. 4b). These hydrogen bonds seem to contribute to the well-ordered conformation of the RDR in the *Ca*THL^V77Q/N153Y/A286K^ mutant. These results indicate that the RDR of the mutant was stabilized, and thus prevented disulfide bond formation under oxidized condition, which consequently resulted in the loss of regulatory mechanism based on redox-switch modulation.

It was notable that the thiolase activity of the *Ca*THL^V77Q/N153Y/A286K^ mutant was more than threefold higher than that of the wild-type enzyme; the activity of the mutant thiolase was similar or even higher than those of the non-redox-regulated thiolases such as *Ec*THL and *Zr*THL (Fig. 3d).

This interesting finding led us to examine the effect of expressing *Ca*THL^V77Q/N153Y/A286K^ mutant on fermentation profile of *C. acetobutylicum*. Two *thlA*-knockdown mutant *C. acetobutylicum* strains overexpressing *Ca*THL and *Ca*THL^V77Q/N153Y/A286K^, respectively, were constructed. When the *thl*_*Ca*_^*V77Q/N153Y/A286K*^ gene was overexpressed in the *thlA-*knockdown mutant under the control of its native promoter, *n*-butanol concentration (7.4 g l^−1^) was higher than that (4.5 g l^−1^) obtained with the *thlA-*knockdown mutant complemented with the *thl*_*Ca*_ (Supplementary Fig. 10). Combined with the results of *in vitro* thiolase activity measurement, these results indicate that redox-regulatory mechanism of *Ca*THL is important in regulating *n*-butanol production in *C. acetobutylicum*. Taken together, the redox-switch regulatory mechanism of *Ca*THL is quite unique, and is responsible for modulating metabolic fluxes at the acetyl-CoA node during the fermentation of *C. acetobutylicum* ATCC 824.

## Discussion

It has been shown that a redox-switch modulation is a general regulatory mechanism in peroxisomal type I degradative thiolases^31^,^32^. However, the mechanism of redox-switch modulation of *Ca*THL is quite different from that of the type I degradative thiolase. In type I degradative thiolase, a disulfide bond is formed between one of the catalytic cysteine residues and a cysteine residue located in domain II of the enzyme. On the other hand, two catalytic cysteine residues are involved in the formation of a disulfide bond in *Ca*THL as described above. The difference in the cysteine residues involved in the disulfide bond formation further causes a big difference in the mode of conformational changes. By the classification based on five *χ* angles of disulfide bonds^33^,^34^, the configuration of the disulfide bond of CaTHL is +/−LHSpiral, which is different from common configurations, such as −RHStaple, −LHHook, and −/+RHHook, found in redox-switch regulated proteins using a reversible disulfide bond^35^. Interestingly, peroxisomal type I degradative thiolase, 3-ketoacyl-CoA thiolase, from *Arabidopsis thaliana* (*At*KAT) showed a −LHSpiral configuration^31^ (Supplementary Fig. 11; Supplementary Table 1).

**Figure 6:**
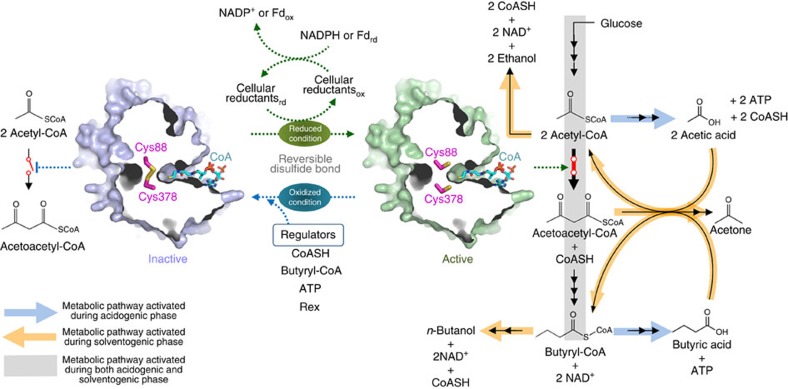
Schematic diagram of redox-switch modulation of *Ca*THL. *Ca*THL catalyses the condensation reaction of acetyl-CoA to acetoacetyl-CoA. In an oxidized status, two catalytic cysteine residues, Cys88 and Cys378, are oxidized forming a disulfide bond, leading to the enzyme inactive and eventually the blockage of the C4 metabolites (*n*-butanol and butyric acid) biosynthesis. When the environment is switched to a reduced status, the disulfide bond is broken and thiol groups of two catalytic residues form an active site environment, enabling the C4 metabolites (*n*-butanol and butyric acid) biosynthesis.

We previously reported that succinic semialdehyde dehydrogenases from human and *E. coli* are controlled by different regulatory mechanisms, redox-switch or non-redox-switch modulation, although they have similar structures and catalytic reactions^36^,^37^,^38^. Combined with the difference in regulatory mechanism of thiolases described above, we propose that enzymes performing the same catalytic reaction could possess different regulatory mechanisms, redox-switch or non-redox-switch modulation, depending on the cellular function of the enzyme and environmental redox potential in which the enzyme is located. Considering that *C. acetobutylicum* is a strict anaerobe and synthesizes *n-*butanol as a main fermentation product through the phase transition from acidogenesis to solventogenesis, it is likely that, unlike aerobic bacteria such as *E. coli* and *Z. ramigera*, it is adapted to have a redox-switch regulated THL for the precise control of the biphasic fermentation (Fig. 6).

For the formation of acetic acid, 1 mol of ATP is generated with 1 mol of CoASH regeneration from 1 mol of acetyl-CoA. On the other hand, only 0.5 mol of ATP is generated with each mole of CoASH and NAD^+^ regeneration via the butyric acid pathway. Thus, formation of the disulfide bond between the two catalytic residues Cys88 and Cys378 in *Ca*THL can be facilitated by intracellular events caused by changing level of CoASH, butyryl-CoA and ATP, while the disulfide bond can be reduced by reactions involving the cellular reductants (Fig. 6). In general, the intracellular redox status is in a reduced state under physiological conditions, which results in free thiols in most cysteine residues^39^. Such intracellular reduced condition often results from the presence of cellular reductants: small peptides with redox-active cysteine residues (thioredoxin and glutaredoxin) and low-molecular-weight thiols (glutathione, glutathionylspermidine, coenzyme B, coenzyme M, mycothiol, ergothioneine, and so on). Interestingly, in the oxidized form of *Ca*THL, the conformational change between the ‘catalytic cysteine loop' and the ‘RDR' forms a hole that enables the cellular reductants to access to the disulfide bond (Supplementary Fig. 12). However, the hole size is not big enough to accommodate *C. acetobutylicum* thioredoxins (105–106 amino acid residues) or glutaredoxin (76 amino acid residues). Thus, the low-molecular-weight thiols can only be accommodated to the hole. Unlike the glutathione system present and well understood in gram-negative bacteria such as *E. coli* and *Alcaligenes faecalis*, *C. acetobutylicum* lacks the genes for synthesizing glutathione. Nevertheless, it has been reported that thiols yet unknown are present at significant levels in clostridia^40^, suggesting that additional novel thiols might be involved in redox regulation of the two catalytic residues Cys88 and Cys378 in *Ca*THL (redox potential of −269.54 mV). Further studies will be needed to identify such novel thiols.

It was interesting to find that non-redox-regulated mutant *Ca*THL^V77Q/N153Y/A286K^, incapable of forming disulfide bond, showed a higher *in vitro* enzyme activity than the wild-type enzyme (Fig. 3d), which resulted in the enhanced butanol production upon its overexpression (Supplementary Fig. 10). In a recent study, *Ca*THL has also been engineered for reduced sensitivity towards its inhibitor CoASH through random mutant library screening^41^. *C. acetobutylicum* ATCC 824 expressing a mutant *Ca*THL containing three amino acid substitutions (R133G, H156N and G222V) showed increased butanol production by 18% compared with the control strain^41^. Taken together, *Ca*THL seems to be responsible for controlling the metabolic fluxes in *C. acetobutylicum* by redox-switch modulation.

Further supporting these findings, overexpression of *Ca*THL in *C. acetobutylicum* ATCC 824 showed a remarkable metabolic shift that disturbed acidogenic to solventogenic transition (Fig. 5; Supplementary Fig. 6); the fermentation profiles were consistent with those reported previously, which showed production of 4.0 g l^−1^ *n*-butanol, 6.7 g l^−1^ butyric acid and 4.1 g l^−1^ acetic acid by the recombinant ATCC 824 strain overexpressing *Ca*THL^42^. Such metabolic shift in *Ca*THL overexpressing strain could simply be restored by co-overexpression of the *adhE1*_*Ca*_ gene encoding aldehyde/alcohol dehydrogenase using the acidogenic *ptb* promoter^42^. Co-overexpression of the *adhE1*_*Ca*_ gene together with the *thl*_*Ca*_ gene during the acidogenic phase could promote early *n*-butanol formation, which led to the normal phase transition. Taken together, these results suggest that the redox-switch modulation indeed affects the *in vivo* activity of *Ca*THL, which consequently alters metabolic flux profiles and the fermentation phase transition as well.

In summary, we have determined the unique crystal structure of *C. acetobutylicum* type II biosynthetic thiolase at 1.77 Å resolution. Unlike other type II biosynthetic thiolases from aerobic bacteria, *C. acetobutylicum* thiolase is regulated by a redox-switch modulation. On the basis of the structural findings together with the results of *in vivo* studies, we propose that the physiological regulation of *C. acetobutylicum* thiolase is mediated by redox-switch modulation during the biphasic fermentation, affecting overall metabolic flux distribution and the acidogenic to solventogenic phase transition. These results will be useful for deciphering fermentation characteristics of *C. acetobutylicum*, and designing metabolic engineering and fermentation strategies for enhanced *n*-butanol production.

## Methods

### Production and purification of thiolases

The gene coding for *C. acetobutylicum* thiolase (*Ca*THL, amino acid residues 1–392) and *E. coli* thiolase (*Ec*THL, amino acid residues 1–394) were amplified from chromosomes of *C. acetobutylicum* and *E. coli* strain by PCR, and the PCR products were then subcloned into pET30a (Novagen) with 6xHis at the C terminus. The resulting expression vectors pET30a:*Ca*THL and pET30a:*Ec*THL were transformed into *E. coli* B834 strain and were grown on LB medium containing 100 mg l^−1^ kanamycin at 37 °C to *A*_600_ of 0.6. After induction with 1.0 mM 1-thio-β-D-galactopyranoside (IPTG) for a further 20 h at 22 °C, the culture was harvested by centrifugation at 5,000*g* for 15 min at 4 °C. The cell pellet was resuspended in ice-cold buffer A (50 mM Tris-HCl, pH 8.0 and 200 mM NaCl) and disrupted by ultrasonication. The cell debris was removed by centrifugation at 11,000*g* for 1 h, and lysate was bound to Ni-NTA agarose column (Qiagen). After washing with buffer A containing 20 mM imidazole, the bound proteins were eluted with 500 mM imidazole in buffer A. Further purification was carried out by applying the HiTrap Q ion exchange chromatography and size exclusion chromatography. The purified proteins were concentrated to 20 g l^−1^ in 50 mM Tris-HCl, pH 8.0 with 200 mM NaCl, and stored at –80 °C for crystallization trials. SDS–polyacrylamide gel electrophoresis analysis of the purified proteins showed a single band of 53.0 and 55.5 kDa that correspond to the calculated molecular weight of *Ca*THL and *Ec*THL monomers, respectively. Site-directed mutagenesis experiments were performed using the QuikChange site-directed mutagenesis kit (Stratagene). The production and purification of the *Ca*THL mutants were carried out by the same procedures as described for the wild-type protein. Primers used for cloning of and site-directed mutagenesis were listed in Supplementary Table 2.

### Crystallization of thiolases

Crystallization of the purified protein was initially performed by the hanging drop vapour diffusion method at 22 °C using commercially available sparse matrix screens from Hampton Research and Emerald BioSystems. Each experiment consisted of mixing 1.5 μl protein solution (20 mg ml^−1^ in 20 mM Tris–HCl pH 8.0, 5 mM β-mercaptoethanol) with 1.5 μl reservoir solution and equilibrating the drop against 0.5 ml reservoir solution. Oxidized form of *Ca*THL crystals were observed from several crystallization screening conditions. After several optimization steps using the hanging drop vapour diffusion method, the best-quality crystals appeared in 5 day using a reservoir solution consisting of 100 mM phosphate-citrate pH 4.2, 10% (wt/vol) polyethylene glycol (PEG) 3,350, 200 mM sodium chloride at 293 K and reached maximal dimensions of ∼0.6 × 0.1 × 0.1 mm. The *Ca*THL^V77Q/N153Y/A286K^ mutant was crystallized from the condition same as the wild-type. The reduced form of *Ca*THL was crystallized using the C378S mutant under the same condition of the oxidized form of the protein except for the addition of 10 mM DTT. For the determination of the *Ca*THL structure in complexed with CoA, the crystals of the *Ca*THL C378S mutant were soaked with 30 mM CoA for 30 min. *Ec*THL crystals obtained with condition of 0.1M acetate pH 5.0 30% PEG400, 0.2 M calcium acetate. For the cryo-protection of both *Ca*THL and *Ec*THL crystals, glycerol of 30% glycerol in reservoir solution was used.

### Data collection and structure determination of thiolases

Data were collected at the 7 Å beamline at the Pohang Accelerator Laboratory using a QUANTUM 270 CCD detector (San Diego, CA, USA) at the wavelength of 1 Å. The oxidized and reduced forms of *Ca*THL crystals diffracted to resolutions of 1.77 and 1.70 Å, respectively. Crystals of CoA-bound form of *Ca*THL and *Ca*THL^V77Q/N153Y/A286K^ mutant diffracted to resolutions of 1.90 and 2.30 Å, respectively. *Ec*THL crystals diffracted to 2.10 Å resolution. The data were then indexed, integrated and scaled using the HKL2000 suite^43^. The oxidized form of *Ca*THL crystals belonged to the space group *P*2_1_2_1_2, with unit cell dimensions of *a*=203.23 Å, *b*=53.99 Å, *c*=72.97 Å and *α*=*β*=*γ*=90°. Assuming two molecules per asymmetric unit, the crystal volume per unit of protein mass was 3.04 Å^3^ Da^−1^, which correspond to a solvent content of ∼59.5% (ref. 44). The reduced and CoA-bound forms of *Ca*THL belonged to the space group *P*2_1_2_1_2 as well, with similar unit cell parameters to those of oxidized form *Ca*THL. The crystal of *Ca*THL^V77Q/N153Y/A286K^ mutant belonged to the space group *C*2 with unit cell dimensions of *a*=116.40 Å, *b*=131.20 Å, *c*=54.12 Å, β=110.30°. With two molecules per asymmetric unit, the crystal volume per unit of protein mass was 2.94 Å^3^ Da^−1^, which correspond to a solvent content of ∼58.2% (ref. 44). *Ec*THL crystals belonged to the space group *P*2_1_2_1_2_1_ and have unit cell dimensions of *a*=74.01 Å, *b*=85.15 Å, *c*=269.11 Å and *α*=*β*=*γ*=90°. Assuming two molecules of *Ec*THL, the crystal volume per unit of protein mass was 3.18 Å^3^ Da^−1^, which correspond to a solvent content of ∼62.4% (ref. 44). The structure of the oxidized form *Ca*THL was solved by molecular replacement method using *MOLREP* (ref. 45) with *Z. ramigera* THL (PDB code 1DLV, 57% amino acid sequence identity) as a search model. The model building was performed manually using the programme *COOT* (ref. 46) and the refinement was performed with CCP4 *REFMAC5* (ref. 47) and *CNS* (ref. 48). The structure of reduced form of *Ca*THL was determined by molecular replacement using *MOLREP* (ref. 45) and the refined oxidized form of *Ca*THL model, and the structures of *Ca*THL^V77Q/N153Y/A286K^ mutant, and CoA-bound *Ca*THL were by the same method and the refined reduced form of *Ca*THL. Structure of *Ec*THL was determined by molecular replacement using *MOLREP* (ref. 45) and the refined reduced form of *Ca*THL model. Refinement of these structures proceeded as for the oxidized form of *Ca*THL. The data statistics are summarized in Table 1. Five refined models of THLs were deposited in the Protein Data Bank.

### Thiolase assay

The spectrophotometric method was used in the assay of thiolase activity by monitoring the increase in absorbance at 303 nm. The reaction mixture contains 67 mM Tris-Cl, pH 8.5, with 5 mM magnesium chloride, 120 μM acetoacetyl-CoA and 300 μM CoA. The reaction was performed at room temperature for 5 min. One unit of enzyme activity is defined as the amount of enzyme catalysing the production of 1 μmol of acetyl-CoA per mg protein per min. To examine the enzyme activity in the various redox environments, 1, 5 and 10 mM of DTT was added to the reaction mixture. To investigate how THL proteins sense redox conditions, fully oxidized THL proteins were treated with various concentrations of H_2_O_2_ for 1 h and enzyme–buffer assay mixture was added. To switch the environment to reduced condition, DTT at 10 mM final concentration was added to H_2_O_2_-treated proteins, and incubated for 30 min. All incubations were performed in triplicates at room temperature. For redox titration experiments, the relative amount of reduced *Ca*THL was measured at 303 nm. Oxidized *Ca*THL (1 μg ml^−1^) was incubated for 2 min in 100 mM 4-(2-hydroxyethyl)-1-piperazineethanesulfonic acid (HEPES; pH 7.0) containing 120 mM acetyl-CoA, 300 mM CoA, and different concentrations of glutathione disulfide (GSSG; 1,000–0 mM) and glutathione (GSH; 0–1,000 mM), generating ambient redox potential of −200 to −320 mV.

### *Clostridium* culture condition for *in vivo* study

*C. acetobutylicum* was cultured in an anaerobic chamber (Coy Laboratory Products, MI) filled with 96% N_2_ and 4% H_2_ gases at 37 °C. Cells were cultured in clostridial growth medium (CGM), which contained per litre: 0.75 g K_2_HPO_4_, 0.75 g KH_2_PO_4_, 0.7 g MgSO_4_·7H_2_O, 0.017 g MnSO_4_·5H_2_O, 0.01 g FeSO_4_·7H_2_O, 2 g (NH_4_)_2_SO_4_, 1 g NaCl, 2 g asparagine, 0.004 g *p*-aminobenzoic acid, 5 g yeast extract and 80 g glucose. Media 2X YTG (pH 5.8, 16 g Bacto tryptone, 10 g yeast extract, 4 g NaCl and 5 g glucose per litre) supplemented with 1.5% agar were used for cell growth on plate.

### Expression of *Ca*THL and *Ca*THL^V77Q/N153Y/A286K^ in *Clostridium*

*E. coli*–*C. acetobutylicum* shuttle vector, pTHL1-Cm was used to clone the *C. acetobutylicum thl* gene. The *thl* gene was amplified with the gDNA of *C. acetobutylicum* ATCC 824 using primers THL_F and THL_R. The PCR products were cloned into the *Pst*I and *Ava*I sites of pTHL1-Cm to construct the *Ca*THL expressing plasmid pTHL1-*Ca*THL. Plasmid expressing *Ca*THL^V77Q/N153Y/A286K^ was constructed based on pTHL1-*Ca*THL by three times of inverse PCR. To make a point mutation at 153th amino acid (N to Y) of the *Ca*THL, PCR was performed with pTHL1-*Ca*THL using primers THL/N153Y_F and THL/N153Y_R. The purified PCR products were ligated by using T4 ligase and T4 polynucleotide kinase at 16 °C. The ligation mixture was transformed into *E. coli* Top 10 (Invitrogen, CA, USA), to screen plasmid harbouring mutated CaTHL^N153Y^. The second (A to K at 286th amino acid) and third (N to Q at 77th amino acid) mutations were constructed in the same method using primer pares THL/A286K_F & R and THL/V77Q_F & R, respectively. Primers used for *in vivo* study of *Ca*THL were listed in Supplementary Table 3.

### *Clostridium* strains and transformation

*C. acetobutylicum* ATCC 824 was kindly provided by Prof. E.T. Papoutsakis (Delaware Biotechnology Institute in University of Delaware, Newark, Delaware, USA). A *thlA*-knockdown mutant *C. acetobutylicum* strain was constructed by integration of the mobile group II intron at the 811th nucleotide in the sense strand. Before transformation of *C. acetobutylicum*, plasmids were methylated to overcome the intracellular restriction system. *E. coli* ER2925 (New England Biolabs, MA, USA) harboring the methylating plasmid pAN1 (ref. 49), which expressed *B. subtilis* phage Φ 51 T1 methyltransferase, was used as a host strain for DNA methylation. Methylated plasmids were transformed into *C. acetobutylicum* strains by an Electro Cell Manipulator (ECM 630, Harvard Apparatus, Holliston, MA, USA; 2.5 kV, 1,575 Ω, 25 μF and 4-mm electrode gap) in the anaerobic chamber. For selecting recombinant strains, erythromycin was used at the final concentration of 40 mg l^−1^.

### Batch fermentation

Batch fermentation was performed anaerobically at 37 °C in a 5-l jar fermentor (Biotron, Korea) containing 1.8 l of CGM. Seed culture of 200 ml was prepared in a 500 ml flask under the anaerobic condition. A single colony was inoculated into a test tube containing 10 ml CGM, grown overnight. The test tube culture was transferred into the flask containing 200 ml fresh CGM. Consequently, cells were inoculated into the bioreactor when the cell density reached the *A*_600_ of 2–3. The culture pH was automatically adjusted above the 5.0 by the addition of 28% (vol/vol) ammonia solution. Antifoam 204 (Sigma-Aldrich, MO, USA) was used to control foaming. Oxygen-free nitrogen gas, obtained by passing it through oxygen trap (Agilent, Germany), was sparged throughout the fermentation at a flow rate of 0.5 l min^−1^. Batch fermentations were performed in duplicates.

### Analysis of metabolites and monitoring cell growth

High performance liquid chromatography (HPLC; Waters 1515/2414/2707, Waters, MA, USA) was used to measure the concentrations of residual glucose and organic acid, such as acetic acid, butyric acid, and lactic acid. Gas chromatography (GC; Agilent 7,890N, Agilent Technologies, CA, USA) was used to detect solvents, such as *n-*butanol, ethanol and acetone. Culture samples were prepared by centrifuged at 7,000*g* for 10 min. The culture supernatant was subjected to GC and HPLC analyses, which were carried out by following the procedures described previously^50^. Cell density was monitored by measuring the *A*_600_ using an Ultrospec 3,000 spectrophotometer (Pharmacia Biotech, Sweden).

### Two-dimensional gel electrophoresis and image analysis

Two-dimensional gel electrophoresis (2-DE) experiments were performed using a PROTEAN IEF Cell (Bio-Rad, Hercules, CA, USA) and a PROTEAN II xi Cell (Bio-Rad), according to the previous work^51^. Protein samples (200 μg) were applied to Immobiline DryStrips (18 cm, pH 4-7; GE Healthcare, NJ, USA) using in-gel rehydration, and applied from 200 to 8,000 V with a total focusing of 60 kV h^−1^. The isofocused strips were electrophoresed on 12% (wt/vol) SDS–polyacrylamide gel electrophoresis gels prepared by the standard protocol^52^. Protein spots were visualized using a silver staining kit (GE Healthcare). The stained gels were scanned by using an UMAX PowerLook 2100XL Scanner (UMAX Technologies, Inc., Dallas, TX, USA). Protein spots were identified by comparing with the proteome reference map of *C. acetobutylicum* reported previously^53^,^54^.

### Metabolic flux analysis

Calculations of *in vivo* fluxes of the *C. acetobutylicum* strains with nonlinear constraints were performed by following the method reported by Desai *et al.*^55^ GAMS (GAMS Corp., Washington, DC, USA) was used for the calculations. The reaction stoichiometry used in this study is described in Supplementary Fig. 5 with details for the nonlinear constraints^55^,^56^.

## Additional information

**Accession codes:** The coordinates and structural factors have been deposited with the Protein Data Bank under accession codes 4XL2 (oxidized form of *Ca*THL), 4XL3 (reduced form of *Ca*THL), 4XL4 (CoA complexed form of *Ca*THL), 4WYR (*Ca*THL^V77Q/N153Y/A286K^ mutant) and 4WYS (*Ec*THL).

**How to cite this article:** Kim, S. *et al.* Redox-switch regulatory mechanism of thiolase from *Clostridium acetobutylicum*. *Nat. Commun.* 6:8410 doi: 10.1038/ncomms9410 (2015).

## Supplementary Material

Supplementary Information Supplementary Figures 1-12 and Supplementary Tables 1-3

## Figures and Tables

**Table 1:** Data collection and refinement statistics of thiolases.

	***Ca*****THL**				***Ec*****THL**
	**Oxidized form**	**Reduced form**	**Complexed with CoA**	**V77Q/N153Y/A286K**	
*Data collection*					
Space group	*P*2_1_2_1_2	*P*2_1_2_1_2	*P*2_1_2_1_2	*C*2	*P*2_1_2_1_2_1_
Cell dimensions					
*a, b, c* (Å)	203.2, 54.0, 73.0	204.4, 54.3, 73.3	204.3, 54.2, 73.1	116.4, 131.2, 54.1	74.0, 85.2, 269.1
*α*, *β*, *γ* (deg)	90.0, 90.0, 90.0	90.0, 90.0, 90.0	90.0, 90.0, 90.0	90.0, 110.3, 90.0	90.0, 90.0, 90.0
Resolution (Å)	50.0–1.77 (1.83-1.77)*	50.0–1.70 (1.73–1.70)*	50.0–1.90 (1.93–1.90)*	50.0–2.30 (2.34–2.30)*	50.0–2.10 (2.14–2.10)*
*R*_sym_	7.0 (29.9)	5.8 (18.0)	9.1 (28.2)	16.6 (30.7)	8.0 (30.6)
*I*/σ*I*	33.7 (2.8)	43.2 (11.3)	33.1 (5.6)	34.3 (10.4)	21.6 (3.9)
Completeness (%)	98.1 (87.9)	97.8 (91.0)	99.9 (99.9)	99.5 (99.5)	99.6 (99.7)
Redundancy	5.3 (4.1)	4.2 (4.0)	7.0 (6.0)	3.9 (3.7)	6.2 (5.4)
					
*Refinement*					
Resolution (Å)	50.0-1.77	50.0-1.70	50.0-1.90	50.0-2.30	50.0-2.10
No. of reflections	73,992	84,226	61,614	31,912	96,899
*R*_work_/*R*_free_	15.7/19.1	15.5/19.2	14.4/18.3	15.3/21.3	17.2/21.8
No. of atoms	6,238	6,622	6,558	6,112	12,024
Protein	5,674	5,800	5,784	5,804	11,332
Ligand/ion	76	36	138	47	0
Water	488	786	636	261	692
*B*-factors	32.2	21.9	24.2	33.2	36.73
Protein	31.2	20.8	23.1	32.6	36.40
Ligand/ion	52.5	33.3	37.9	55.9	0
Water	39.4	32.9	33.6	36.9	39.47
R.m.s.d.					
Bond length (Å)	0.018	0.022	0.018	0.016	0.016
Bond angles (deg)	1.811	2.031	1.886	1.703	1.789

^*^Highest-resolution shell is shown in parentheses.

## References

[b1] Mitchell W. J. Physiology of carbohydrate to solvent conversion by clostridia. Adv. Microb. Physiol. 39, 31–130 (1998). 9328646 10.1016/s0065-2911(08)60015-6

[b2] Papoutsakis E. T. Engineering solventogenic clostridia. Curr. Opin. Biotechnol. 19, 420–429 (2008). 10.1016/j.copbio.2008.08.003 18760360

[b3] Inui M. *et al.* Expression of Clostridium acetobutylicum butanol synthetic genes in *Escherichia coli*. Appl. Microbiol. Biotechnol. 77, 1305–1316 (2008). 10.1007/s00253-007-1257-5 18060402

[b4] Durre P. Fermentative butanol production: bulk chemical and biofuel. Ann. NY Acad. Sci. 1125, 353–362 (2008). 18378605 10.1196/annals.1419.009

[b5] Durre P. Biobutanol: an attractive biofuel. Biotechnol. J. 2, 1525–1534 (2007). 10.1002/biot.200700168 17924389

[b6] Lee S. Y. *et al.* Fermentative butanol production by clostridia. Biotechnol. Bioeng. 101, 209–228 (2008). 18727018 10.1002/bit.22003

[b7] Jones D. T. & Woods D. R. Acetone-butanol fermentation revisited. Microbiol. Rev. 50, 484–524 (1986). 3540574 10.1128/mr.50.4.484-524.1986 PMC373084

[b8] Ezeji T., Milne C., Price N. D. & Blaschek H. P. Achievements and perspectives to overcome the poor solvent resistance in acetone and butanol-producing microorganisms. Appl. Microbiol. Biotechnol. 85, 1697–1712 (2010). 20033401 10.1007/s00253-009-2390-0

[b9] Atsumi S., Hanai T. & Liao J. C. Non-fermentative pathways for synthesis of branched-chain higher alcohols as biofuels. Nature 451, 86–89 (2008). 18172501 10.1038/nature06450

[b10] Steen E. J. *et al.* Metabolic engineering of *Saccharomyces cerevisiae* for the production of n-butanol. Microb. Cell Fact. 7, 36 (2008). 19055772 10.1186/1475-2859-7-36 PMC2621116

[b11] Nielsen D. R. *et al.* Engineering alternative butanol production platforms in heterologous bacteria. Metab. Eng. 11, 262–273 (2009). 19464384 10.1016/j.ymben.2009.05.003

[b12] Lee S. K., Chou H., Ham T. S., Lee T. S. & Keasling J. D. Metabolic engineering of microorganisms for biofuels production: from bugs to synthetic biology to fuels. Curr. Opin. Biotechnol. 19, 556–563 (2008). 18996194 10.1016/j.copbio.2008.10.014

[b13] Felnagle E. A., Chaubey A., Noey E. L., Houk K. N. & Liao J. C. Engineering synthetic recursive pathways to generate non-natural small molecules. Nat. Chem. Biol. 8, 518–526 (2012). 22596203 10.1038/nchembio.959

[b14] Modis Y. & Wierenga R. K. A biosynthetic thiolase in complex with a reaction intermediate: the crystal structure provides new insights into the catalytic mechanism. Structure 7, 1279–1290 (1999). 10545327 10.1016/s0969-2126(00)80061-1

[b15] Mathieu M. *et al.* The 2.8A crystal structure of peroxisomal 3-ketoacyl-CoA thiolase of *Saccharomyces cerevisiae*: a five-layered alpha beta alpha beta alpha structure constructed from two core domains of identical topology. Structure 2, 797–808 (1994). 7812714 10.1016/s0969-2126(94)00081-6

[b16] Mathieu M. *et al.* The 1.8A crystal structure of the dimeric peroxisomal 3-ketoacyl-CoA thiolase of *Saccharomyces cerevisiae*: implications for substrate binding and reaction mechanism. J. Mol. Biol. 273, 714–728 (1997). 9402066 10.1006/jmbi.1997.1331

[b17] Meriläinen G., Poikela V., Kursula P. & Wierenga R. K. The thiolase reaction mechanism: the importance of Asn316 and His348 for stabilizing the enolate intermediate of the Claisen condensation. Biochemistry 48, 11011–11025 (2009). 19842716 10.1021/bi901069h

[b18] Kursula P., Sikkila H., Fukao T., Kondo N. & Wierenga R. K. High resolution crystal structures of human cytosolic thiolase (CT): a comparison of the active sites of human CT, bacterial thiolase, and bacterial KAS I. J. Mol. Biol. 347, 189–201 (2005). 15733928 10.1016/j.jmb.2005.01.018

[b19] Meriläinen G., Schmitz W., Wierenga R. K. & Kursula P. The sulfur atoms of the substrate CoA and the catalytic cysteine are required for a productive mode of substrate binding in bacterial biosynthetic thiolase, a thioester-dependent enzyme. FEBS J. 275, 6136–6148 (2008). 19016856 10.1111/j.1742-4658.2008.06737.x

[b20] Modis Y. & Wierenga R. K. Crystallographic analysis of the reaction pathway of *Zoogloea ramigera* biosynthetic thiolase. J. Mol. Biol. 297, 1171–1182 (2000). 10764581 10.1006/jmbi.2000.3638

[b21] Barford D. The role of cysteine residues as redox-sensitive regulatory switches. Curr. Opin. Struct. Biol. 14, 679–686 (2004). 15582391 10.1016/j.sbi.2004.09.012

[b22] Choi H. J. *et al.* Structural basis of the redox switch in the OxyR transcription factor. Cell 105, 103–113 (2001). 11301006 10.1016/s0092-8674(01)00300-2

[b23] Ilbert M. *et al.* The redox-switch domain of Hsp33 functions as dual stress sensor. Nat. Struct. Mol. Biol. 14, 556–563 (2007). 17515905 10.1038/nsmb1244 PMC2782886

[b24] Wiesenborn D. P., Rudolph F. B. & Papoutsakis E. T. Thiolase from *Clostridium acetobutylicum* ATCC 824 and its role in the synthesis of acids and solvents. Appl. Environ. Microbiol. 54, 2717–2722 (1988). 16347774 10.1128/aem.54.11.2717-2722.1988 PMC204361

[b25] Zhao Y., Tomas C. A., Rudolph F. B., Papoutsakis E. T. & Bennett G. N. Intracellular butyryl phosphate and acetyl phosphate concentrations in *Clostridium acetobutylicum* and their implications for solvent formation. Appl. Environ. Microbiol. 71, 530–537 (2005). 15640230 10.1128/AEM.71.1.530-537.2005 PMC544202

[b26] Harris L. M., Desai R. P., Welker N. E. & Papoutsakis E. T. Characterization of recombinant strains of the *Clostridium acetobutylicum* butyrate kinase inactivation mutant: need for new phenomenological models for solventogenesis and butanol inhibition? Biotechnol. Bioeng. 67, 1–11 (2000). 10581430

[b27] Husemann M. H. & Papoutsakis E. T. Solventogenesis in *Clostridium acetobutylicum* fermentations related to carboxylic acid and proton concentrations. Biotechnol. Bioeng. 32, 843–852 (1988). 18587795 10.1002/bit.260320702

[b28] Speakman H. B. Gas production during the acetone and butyl alcohol fermentation of starch. J. Biol. Chem. 43, 401–411 (1920).

[b29] Millat T. *et al.* A shift in the dominant phenotype governs the pH-induced metabolic switch of *Clostridium acetobutylicumin* phosphate-limited continuous cultures. Appl. Microbiol. Biotechnol. 97, 6451–6466 (2013). 23640360 10.1007/s00253-013-4860-7

[b30] Millat T., Janssen H., Bahl H., Fischer R. J. & Wolkenhauer O. Integrative modelling of pH-dependent enzyme activity and transcriptomic regulation of the acetone-butanol-ethanol fermentation of *Clostridium acetobutylicum* in continuous culture. Microb. Biotechnol. 6, 526–539 (2013). 23332010 10.1111/1751-7915.12033 PMC3918155

[b31] Sundaramoorthy R. *et al.* The crystal structure of a plant 3-ketoacyl-CoA thiolase reveals the potential for redox control of peroxisomal fatty acid beta-oxidation. J. Mol. Biol. 359, 347–357 (2006). 16630629 10.1016/j.jmb.2006.03.032

[b32] Pye V. E., Christensen C. E., Dyer J. H., Arent S. & Henriksen A. Peroxisomal plant 3-ketoacyl-CoA thiolase structure and activity are regulated by a sensitive redox switch. J. Biol. Chem. 285, 24078–24088 (2010). 20463027 10.1074/jbc.M110.106013 PMC2911321

[b33] Schmidt B., Ho L. & Hogg P. J. Allosteric disulfide bonds. Biochemistry 45, 7429–7433 (2006). 16768438 10.1021/bi0603064

[b34] Harrison P. M. & Sternberg M. J. The disulphide beta-cross: from cystine geometry and clustering to classification of small disulphide-rich protein folds. J. Mol. Biol. 264, 603–623 (1996). 8969308 10.1006/jmbi.1996.0664

[b35] Hogg P. J. Targeting allosteric disulphide bonds in cancer. Nat. Rev. Cancer 13, 425–431 (2013). 23660784 10.1038/nrc3519

[b36] Kim Y.-G. *et al.* Redox-switch modulation of human SSADH by dynamic catalytic loop. EMBO J. 28, 959–968 (2009). 19300440 10.1038/emboj.2009.40 PMC2670868

[b37] Ahn J. W., Kim Y. G. & Kim K. J. Crystal structure of non-redox regulated SSADH from *Escherichia coli*. Biochem. Biophys. Res. Commun. 392, 106–111 (2010). 20060383 10.1016/j.bbrc.2010.01.014

[b38] Kim K.-J. *et al.* Succinic semialdehyde dehydrogenase: biochemical–molecular–clinical disease mechanisms, redox regulation, and functional significance. Antioxid. Redox Signal. 15, 691–718 (2011). 20973619 10.1089/ars.2010.3470 PMC3125545

[b39] Bardwell J. C. Building bridges: disulphide bond formation in the cell. Mol. Microbiol. 14, 199–205 (1994). 7830566 10.1111/j.1365-2958.1994.tb01281.x

[b40] Newton G. & Fahey R. Glutathione in procaryotes CRC Press (1989).

[b41] Mann M. S. & Lütke-Eversloh T. Thiolase engineering for enhanced butanol production in *Clostridium acetobutylicum*. Biotechnol. Bioeng. 110, 887–897 (2013). 23096577 10.1002/bit.24758

[b42] Sillers R., Al-Hinai M. A. & Papoutsakis E. T. Aldehyde–alcohol dehydrogenase and/or thiolase overexpression coupled with CoA transferase downregulation lead to higher alcohol titers and selectivity in *Clostridium acetobutylicum* fermentations. Biotechnol. Bioeng. 102, 38–49 (2009). 18726959 10.1002/bit.22058

[b43] Otwinowski Z. & Minor W. Processing of X-ray diffraction data. Methods Enzymol. 276, 307–326 (1997). 10.1016/S0076-6879(97)76066-X 27754618

[b44] Matthews B. W. Solvent content of protein crystals. J. Mol. Biol. 33, 491–497 (1968). 5700707 10.1016/0022-2836(68)90205-2

[b45] Vagin A. & Teplyakov A. Molecular replacement with MOLREP. Acta Crystallogr. D Biol. Crystallogr. 66, 22–25 (2010). 20057045 10.1107/S0907444909042589

[b46] Emsley P. & Cowtan K. Coot: model-building tools for molecular graphics. Acta Crystallogr. D Biol. Crystallogr. 60, 2126–2132 (2004). 15572765 10.1107/S0907444904019158

[b47] Murshudov G. N., Vagin A. A. & Dodson E. J. Refinement of macromolecular structures by the maximum-likelihood method. Acta Crystallogr. D Biol. Crystallogr. 53, 240–255 (1997). 15299926 10.1107/S0907444996012255

[b48] Brunger A. T. *et al.* Crystallography & NMR system: a new software suite for macromolecular structure determination. Acta Crystallogr. D Biol. Crystallogr. 54, 905–921 (1998). 9757107 10.1107/s0907444998003254

[b49] Mermelstein L. D. & Papoutsakis E. T. In vivo methylation in *Escherichia coli* by the *Bacillus subtilis* phage phi 3T I methyltransferase to protect plasmids from restriction upon transformation of *Clostridium acetobutylicum* ATCC 824. Appl. Environ. Microbiol. 59, 1077–1081 (1993). 8386500 10.1128/aem.59.4.1077-1081.1993 PMC202241

[b50] Jang Y. S., Im J. A., Choi S. Y., Lee J. I. & Lee S. Y. Metabolic engineering of *Clostridium acetobutylicum* for butyric acid production with high butyric acid selectivity. Metab. Eng. 23, 165–174 (2014). 24704310 10.1016/j.ymben.2014.03.004

[b51] Han M.-J., Jeong K. J., Yoo J.-S. & Lee S. Y. Engineering *Escherichia coli* for increased productivity of serine-rich proteins based on proteome profiling. Appl. Environ. Microbiol. 69, 5772–5781 (2003). 14532024 10.1128/AEM.69.10.5772-5781.2003 PMC201230

[b52] Laemmli U. K. Cleavage of structural proteins during the assembly of the head of bacteriophage T4. Nature 227, 680–685 (1970). 5432063 10.1038/227680a0

[b53] Jang Y.-S. *et al.* Proteomic analyses of the phase transition from acidogenesis to solventogenesis using solventogenic and non-solventogenic *Clostridium acetobutylicum* strains. Appl. Microbiol. Biotechnol. 98, 5105–5115 (2014). 24743985 10.1007/s00253-014-5738-z

[b54] Mao S. *et al.* Proteome reference map and comparative proteomic analysis between a wild type *Clostridium acetobutylicum* DSM 1731 and its mutant with enhanced butanol tolerance and butanol yield. J. Proteome Res. 9, 3046–3061 (2010). 20426490 10.1021/pr9012078

[b55] Desai R. P., Nielsen L. K. & Papoutsakis E. T. Stoichiometric modeling of *Clostridium acetobutylicum* fermentations with non-linear constraints. J. Biotechnol. 71, 191–205 (1999). 10483106 10.1016/s0168-1656(99)00022-x

[b56] Jang Y. S. *et al.* Enhanced butanol production obtained by reinforcing the direct butanol-forming route in *Clostridium acetobutylicum*. mBio 3, e00314–12 (2012). 23093384 10.1128/mBio.00314-12 PMC3482502

