# Supplementary material for: Redox-switch regulatory mechanism of thiolase from Clostridium acetobutylicum
Source: Nat Commun. 2015 Sep 22;6:8410. doi: 10.1038/ncomms9410 (PMC4595758; doi:10.1038/ncomms9410)
Supplement: Supplementary Information — Supplementary Figures 1-12 and Supplementary Tables 1-3 [file ncomms9410-s1.pdf]

Supplementary Materials

Redox-switch regulatory mechanism of thiolase from *Clostridium acetobutylicum*

Sangwoo Kim, Yu-Sin Jang, Sung-Chul Ha, Jae-Woo Ahn, Eun-Jung Kim, Jae Hong Lim, Changhee Cho, Yong Shin Ryu, Sung Kuk Lee, Sang Yup Lee* & Kyung-Jin Kim*

*Correspondence should be addressed to: Email: kkim@knu.ac.kr
leesy@kaist.ac.kr

This PDF file includes:

Supplementary Figures 1 to 12 and Supplementary Table 1 to 3.

a

b

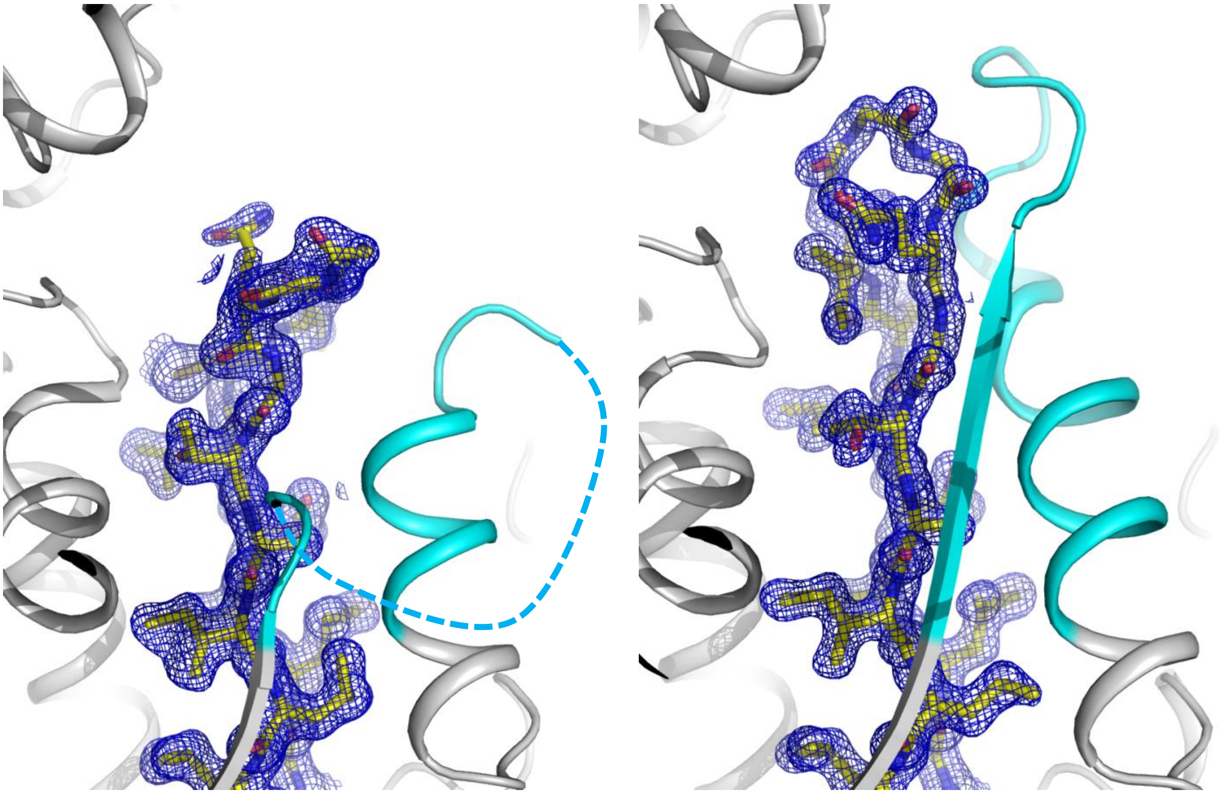

Supplementary Figure 1. Electron density map of the catalytic cysteine loop. (a) Electron density map of the catalytic cysteine loop in the oxidized form of *Ca*THL. The the oxidized form of *Ca*THL is shown as a cartoon diagram. The catalytic cysteine loop is distinguished as a stick model with yellow color. The RDR is shown with cyan color. The electron density map is contoured at 2.0 σ. (b) Electron density map of the catalytic cysteine loop in the reduced form of *Ca*THL. The catalytic cysteine loop and the RDR are presented as in (a).

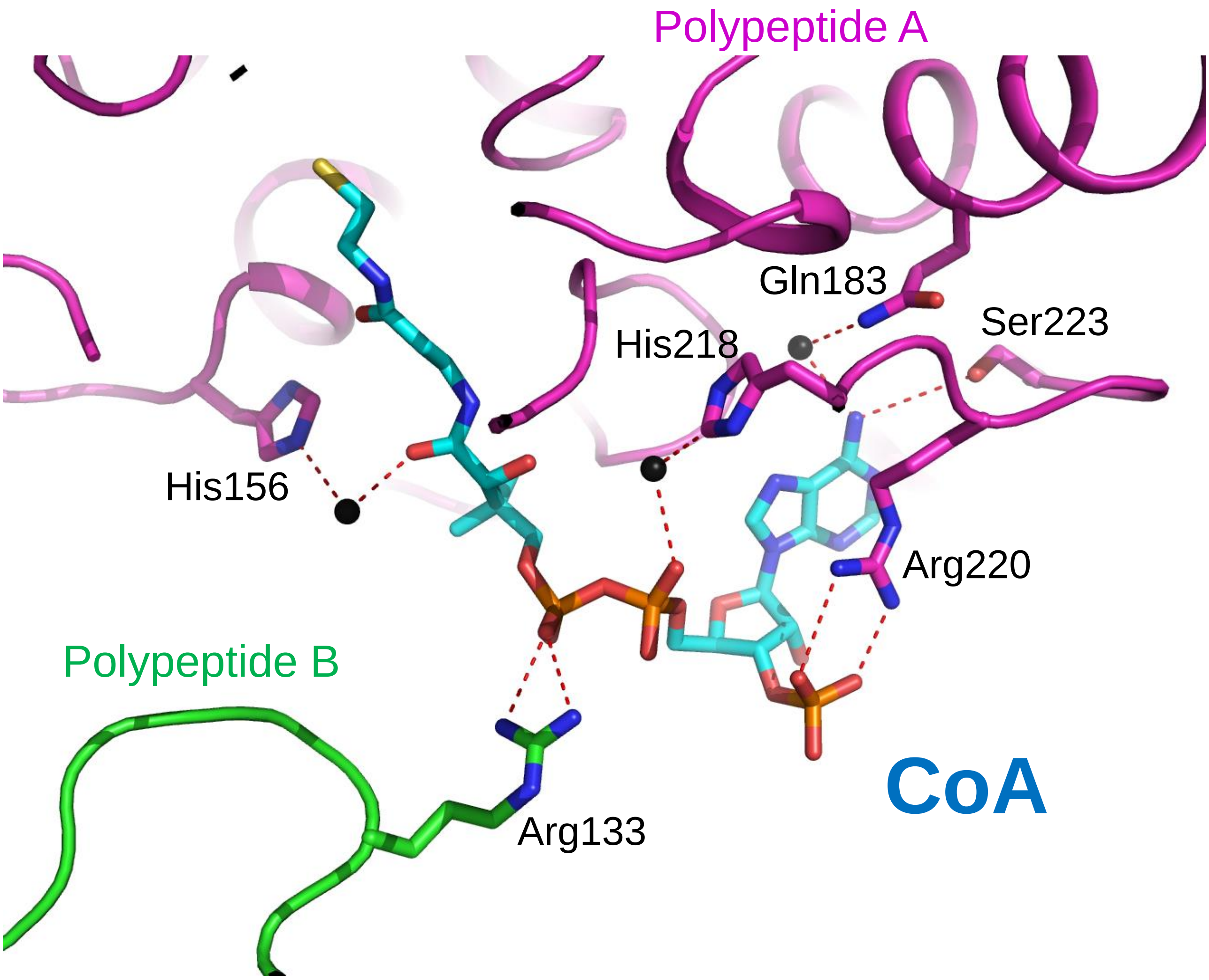

Supplementary Figure 2. CoA binding mode of *Ca*THL. The residues involved in hydrogen bond formations with CoA are shown as a stick model. His156, Gln183, His218, Arg220 and Ser223 residues of one polypeptide are shown as magentas color, and Arg133 residue of another polypeptide is as green color. Water molecules involved in the stabilization of CoA are shown by black spheres. The hydrogen bond interactions are indicated by red dotted lines.

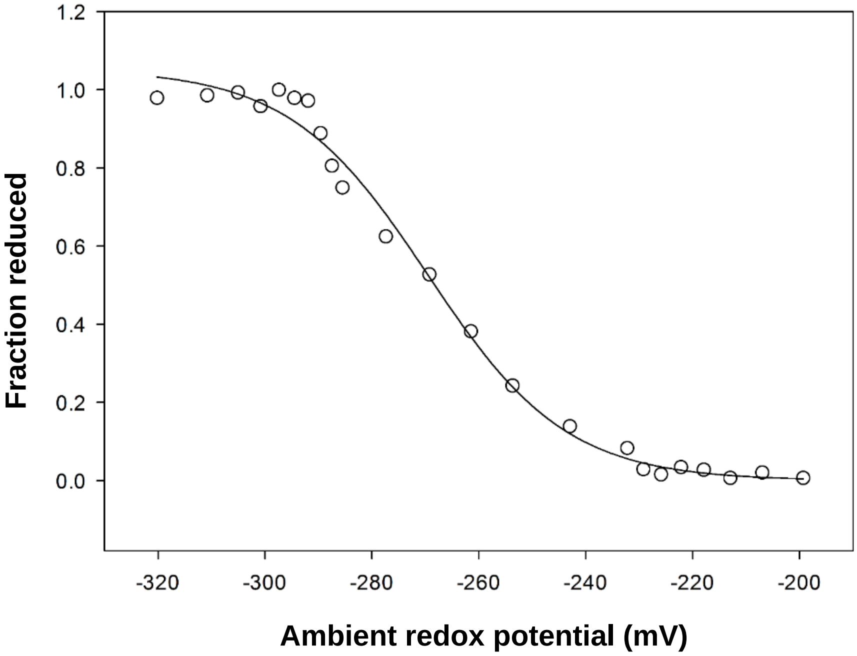

Supplementary Figure 3. Determination of the redox potential of the disulfide in *Ca*THL. The relative amount of reduced *Ca*THL was measured at 303 nm. Oxidized *Ca*THL (1 μg ml^{-1}) was incubated for 2 min in 100 mM HEPES (pH 7.0) containing 120 μM acetyl-CoA, 300 μM CoA and different concentrations of glutathione disulfide (GSSG; 1000 to 0 μM) and glutathione (GSH; 0 to 1000 μM), generating ambient redox potentials of -200 to -320 mV. The solid line represents the best fit with the midpoint potential value of -269.54 mV (n=2).

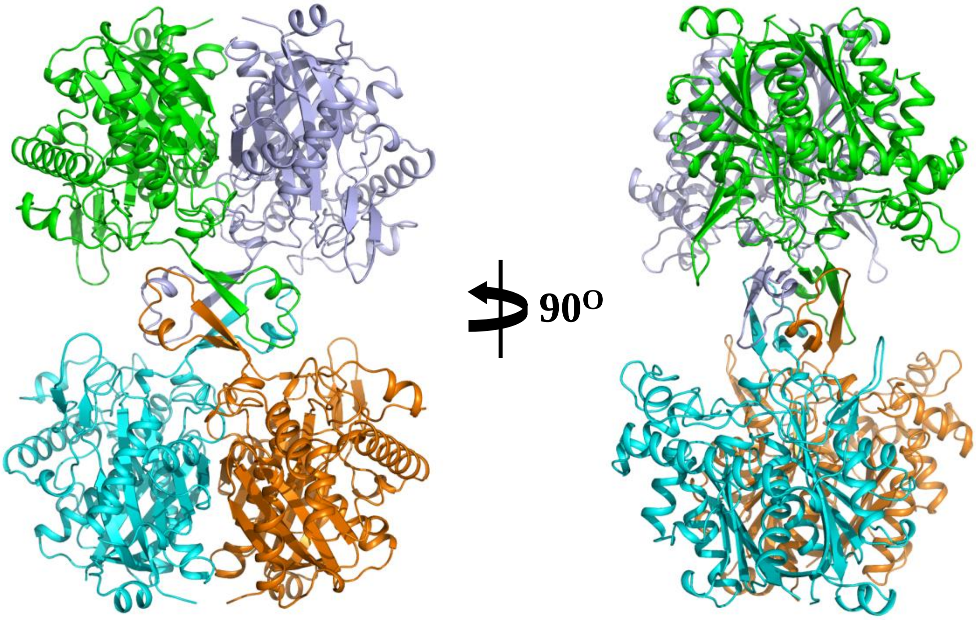

Supplementary Figure 4. Crystal structure of *Ec*THL (AtoB). A tetrameric protein is shown as ribbon representation in which four momomers are distinguished as green, light blue, cyan and orange colors, respectively.

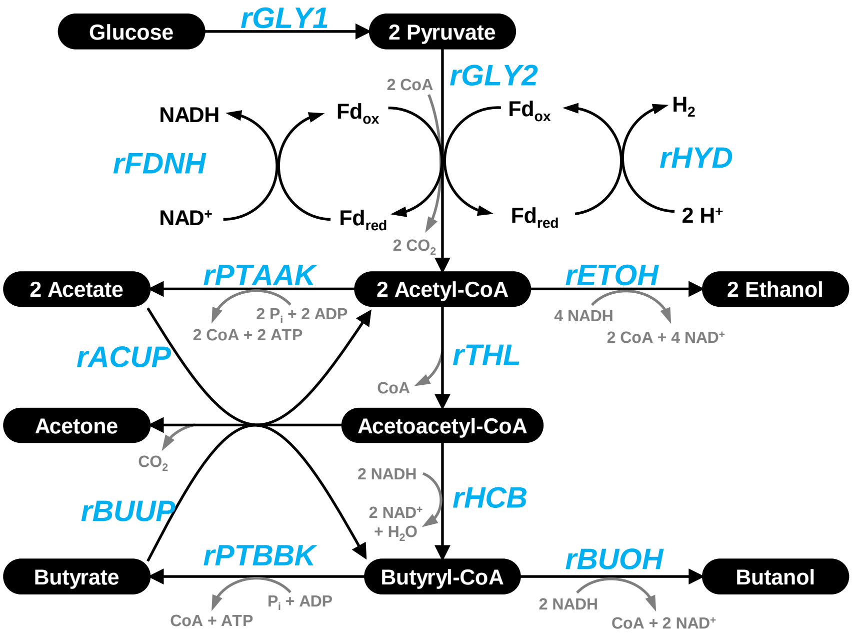

rBIO	Glucose + 0.873 NADH + xATP ↔ 6 Biomass
rGLY1	Glucose → 2 Pyruvate + 2 NADH + 2 ATP
rGLY2	Pyruvate → Acetyl-CoA + CO_2 + FdRed
rETOH	Acetyl-CoA + 2 NADH → Ethanol
rPTAAK	Acetyl-CoA ↔ Acetate + ATP
rTHL	2 Acetyl-CoA ↔ Acetoacetyl-CoA
rACUP	Acetoacetyl-CoA + Acetate ↔ Acetone + CO_2 + Acetyl-CoA
rBYUP	Acetoacetyl-CoA + Butyrate ↔ Acetone + CO_2 + Butyryl-CoA
rBYCA	Acetoacetyl-CoA + 2 NADH ↔ Butyryl-CoA
rPTBBK	Butyryl-CoA ↔ Butyrate + ATP
rBUOH	Butyryl-CoA + 2 NADH → Butanol
rHYD	FdRed → H_2
rFDNH	FdRed ↔ NADH

Supplementary Figure 5. Schematic diagram of the model used in this study for metabolic flux analysis of *C. acetobutylicum*. Cyan color presents the specific flux. Reactions in the yellow box present stoichiometry for metabolic flux analysis. Metabolic flux analyses were minimization of the following equation: $\|\mathbf{W}^{-1}\mathbf{A}\mathbf{r} - \mathbf{W}^{-1}\mathbf{x}\|^2 + (\text{rBYUP}[\text{acetate}]_e - 0.315\text{rACUP}[\text{butyrate}]_e)^2$, subject to $\mathbf{l} \leq \mathbf{r} \leq \mathbf{u}$, where **A** is the stoichiometric matrix, **r** is the flux vector, and **x** is the vector determining the accumulation of metabolite. The vectors **l** and **u** constrain the lower and upper limits of **r**, respectively. **W** is a diagonal weighting matrix. For species existing only in the intracellular space, the weighting factor determines the scale of accumulation level. For measured extracellular species, the entries of **W** are the standard deviations of measurement.

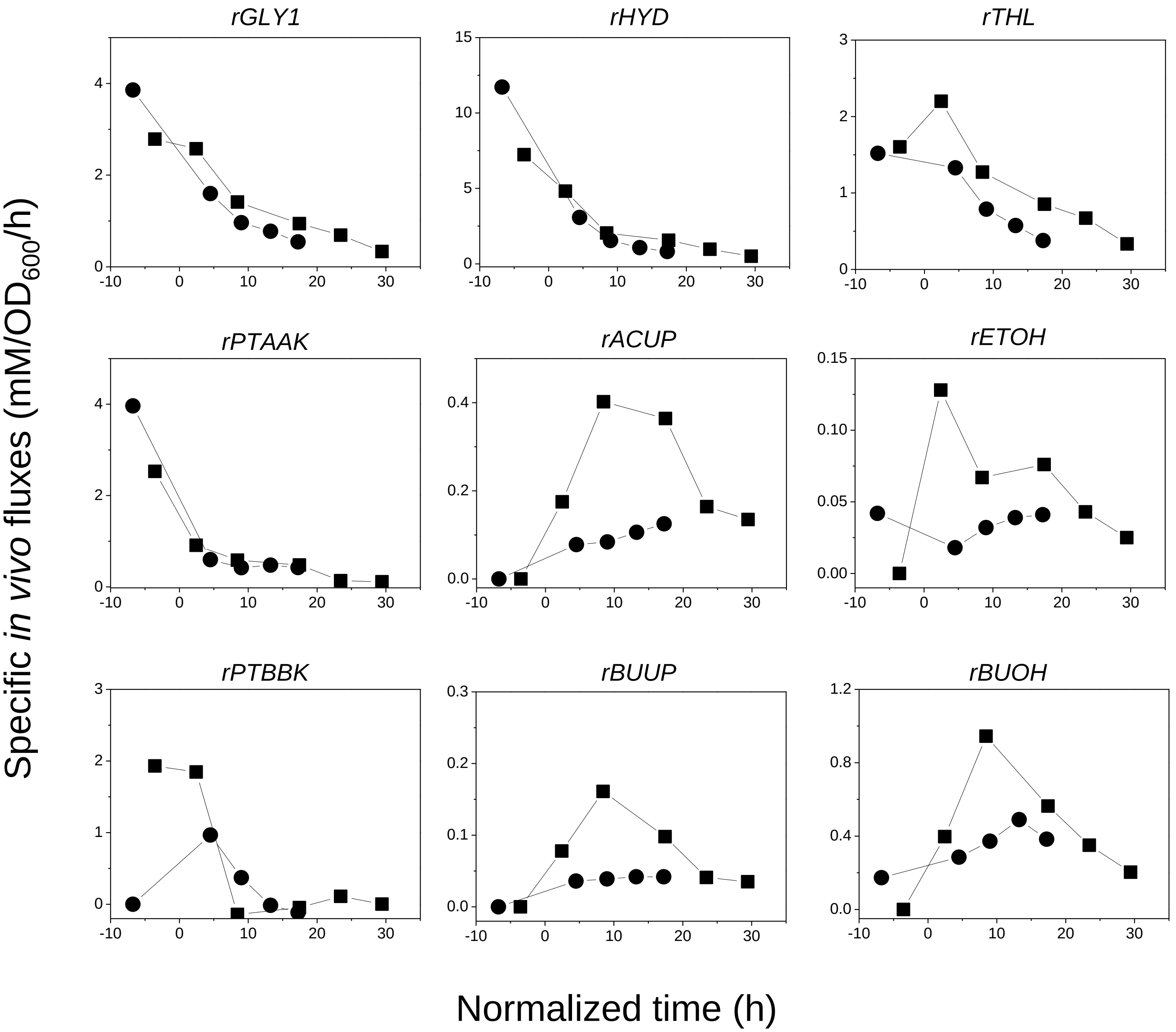

Supplementary Figure 6. Calculation of internal fluxes of the *C. acetobutylicum* ATCC 824 with (closed circles) and without (closed squares) overexpression of *Ca*THL. Differences of glucose and product concentrations between one sampling point and the next sampling point were used to obtain flux profile of an interval. Lag-time of the fermentations was normalized by considering the time at OD_{600} of 1.0 as zero. See Supplementary Figure 5 for schematic diagram of the model used in this study for metabolic flux analysis.

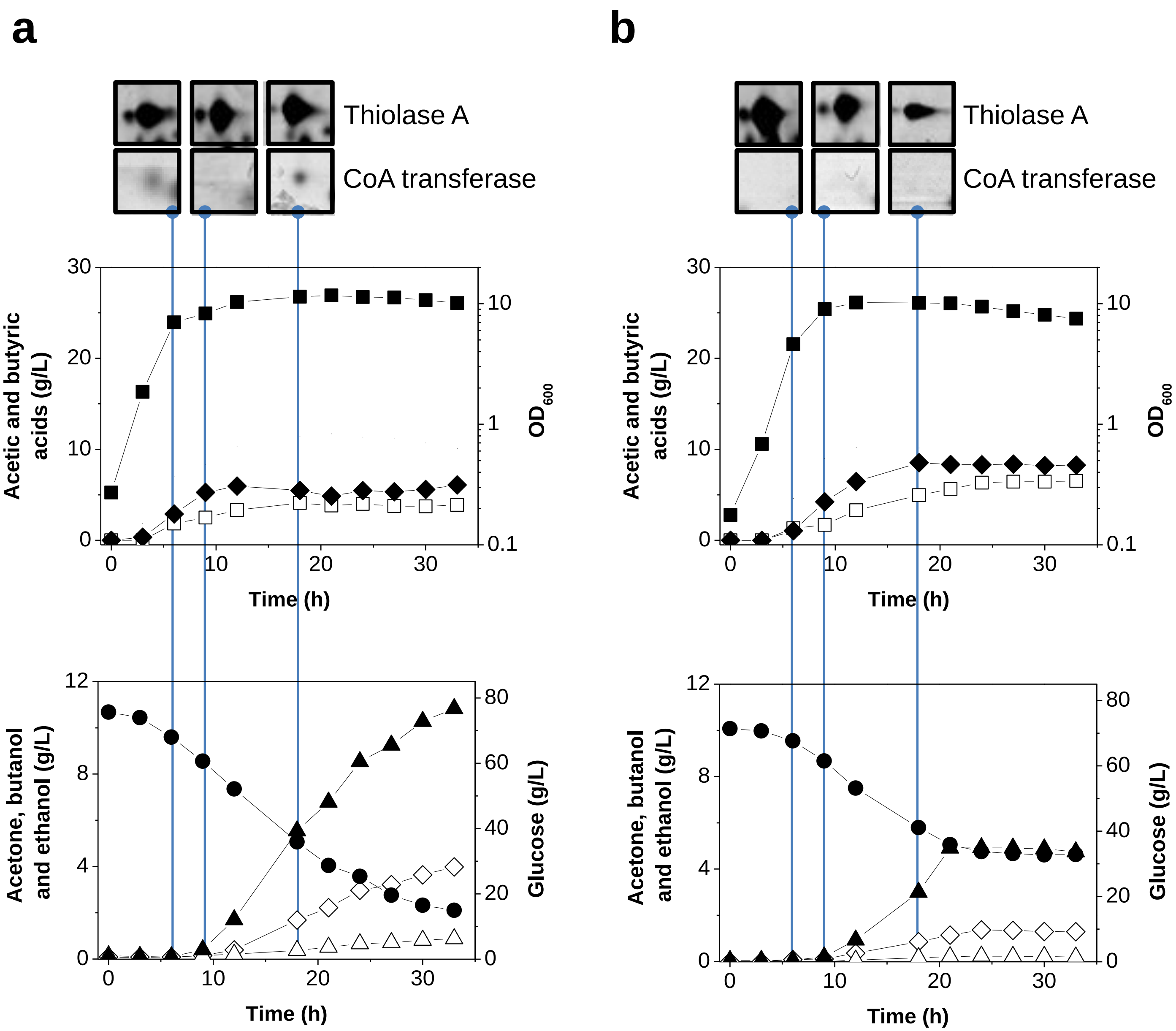

Supplementary Figure 7. Anaerobic batch fermentation profiles of the biphasic *C. acetobutylicum* ATCC 824 (a) and the ATCC 824 strain overexpressing the CaTHL (b) are shown. Proteome samples were collected at culture time of 6, 9, and 18 h, which are presenting acidogenic, early solventogenic, and solventogenic phases, respectively. Protein sports corresponding to thiolase and CoA transferase are captured from the 2D-gel (Supplementary Fig. 8) and displayed with the fermentation profiles. Symbols are: acetic acid (open squares), butyric acid (filled diamonds), OD600 (filled squares), glucose (filled circles), acetone (open diamonds), ethanol (open triangles) and n-butanol (closed triangles). Fermentations were performed at least in duplicates showing reproducibility, and the profiles shown are that of one representative fermentation.

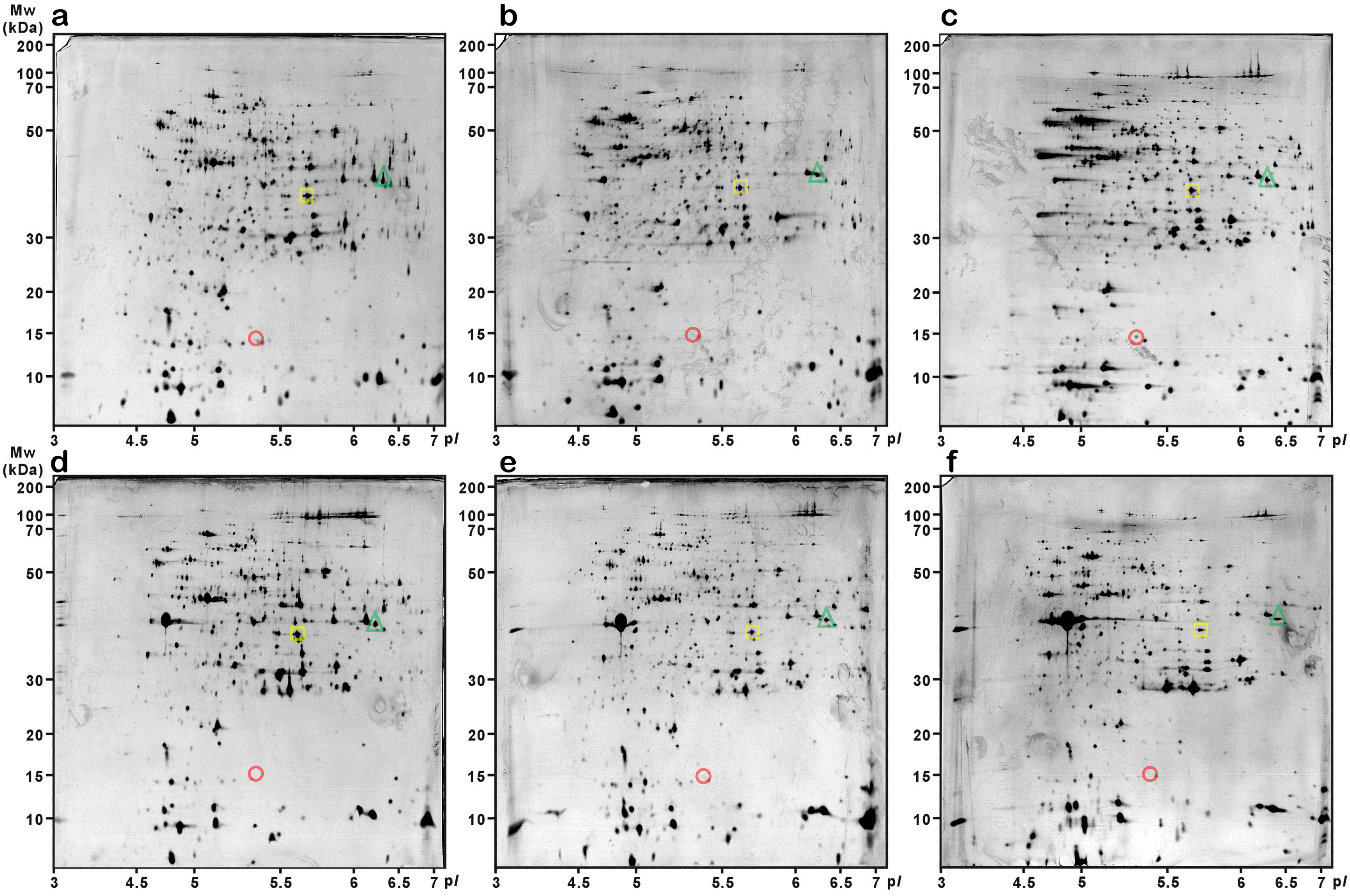

Supplementary Figure 8. Representative 2-DE gels of the *thlA*-knockdown mutant *C. acetobutylicum* strains overexpressing *Ca*THL (a-c) and *Ca*THL$^{V77Q/N153Y/A286K}$ (d-e), individually. Proteome samples were collected at culture time of 6 h (a and d), 9 h (b and e), and 18 h (c and f), which are presenting acidogenic, early solventogenic, and solventogenic phases, respectively (see Supplementary Figure 7 for fermentation profiles). Spots indicated by red circle, yellow square, and green triangle are presenting CoA transferase, thiolase, and butyrate kinase, respectively.

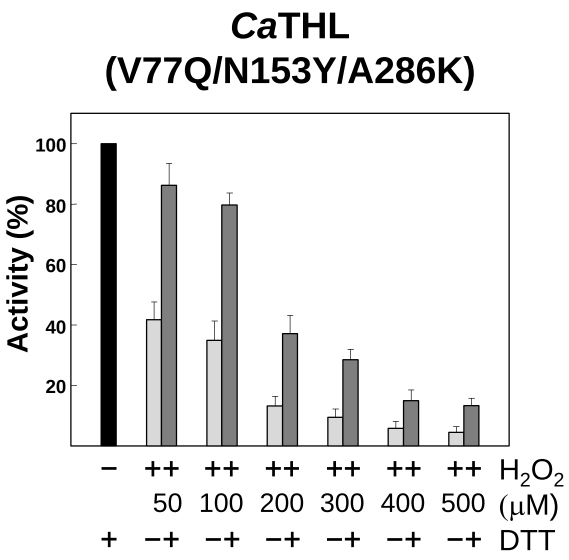

Supplementary Figure 9. Activity recovery test of *Ca*THL$^{V77Q/N153Y/A286K}$ mutant . To investigate how *Ca*THL mutant senses an oxidative environment, and confirm no disulfide bond is formed in the *Ca*THL mutant under oxidized condition, the mutant protein was treated with various concentrations of H_2O_2 and the thiolase activities were measured. We then added 10 mM DTT to switch the environment to a reduced state, and the thiolase activities were measured. Unlike the wild-type *Ca*THL in which over 80% of activity was recovered from the reaction mixture that was treated with even 500 mM H_2O_2, almost no activities were recovered from the mutant that was treated with more than 400 mM H_2O_2. The results indicate that the *Ca*THL$^{V77Q/N153Y/A286K}$ mutant do not form a disulfide bond even under the oxidation condition, and the regulatory mechanism of the mutant protein is switched from the redox-switch modulation to the non-redox switch modulation.

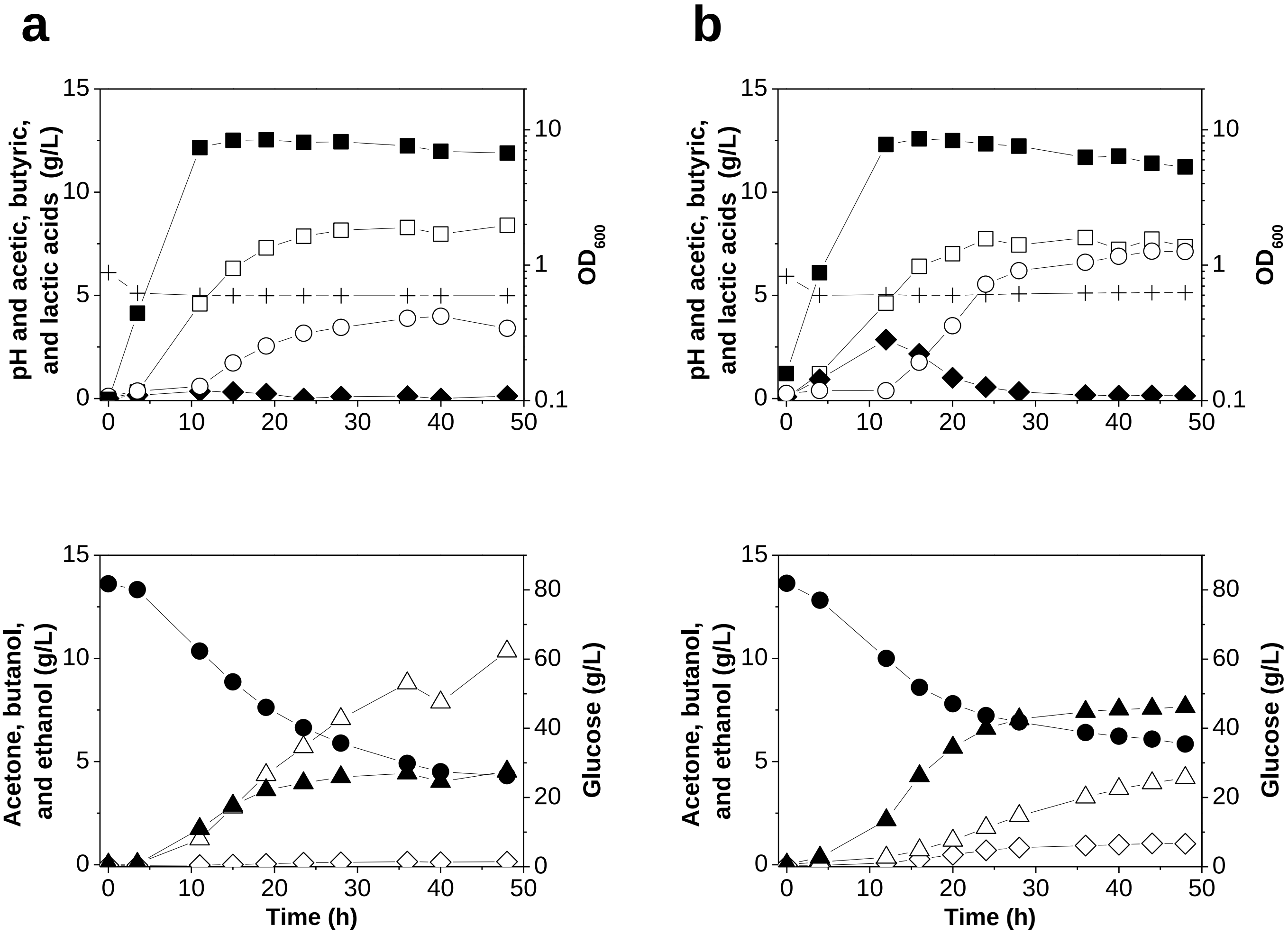

Supplementary Figure 10. Anaerobic batch fermentation profiles of the *thlA*-knockdown mutant *C. acetobutylicum* strains overexpressing *Ca*THL (a) and *Ca*THL$^{V77Q/N153Y/A286K}$ (b), individually. Symbols are: acetic acid (open squares), butyric acid (filled diamonds), lactic acid (open circles), pH (crosses), OD600 (filled squares), glucose (filled circles), acetone (open diamonds), ethanol (open triangles) and *n*-butanol (closed triangles). Fermentations were performed at least in duplicates showing reproducibility, and the profiles shown are that of one representative fermentation.

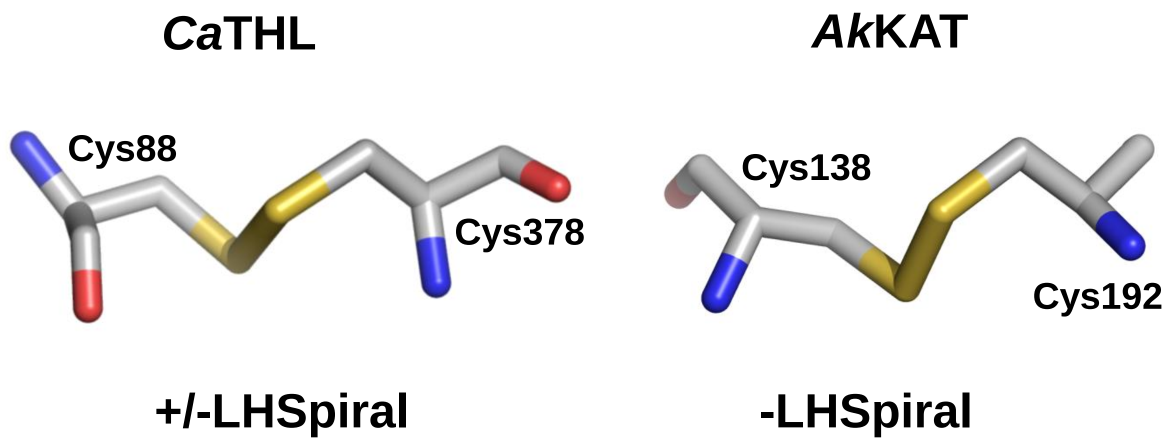

Supplementary Figure 11. Comparison of the configurations of the disulfide bonds of *Ca*THL and *Ak*KAT. The stick models of the disulfide bonds of *Ca*THL and *Ak*THL (PDB ID; 2C7Y), both in the oxidized form, are shown (nitrogen, blue; oxygen, red; carbon, light grey; sulfur, yellow). The analysis with online tool (Disulfide Bond Analysis) showed that the configurations of the disulfide bonds of *Ca*THL and *Ak*THL are +/-LHSpiral and –LHSpiral, respectively, which is not common emerging allosteric disulfide bond.

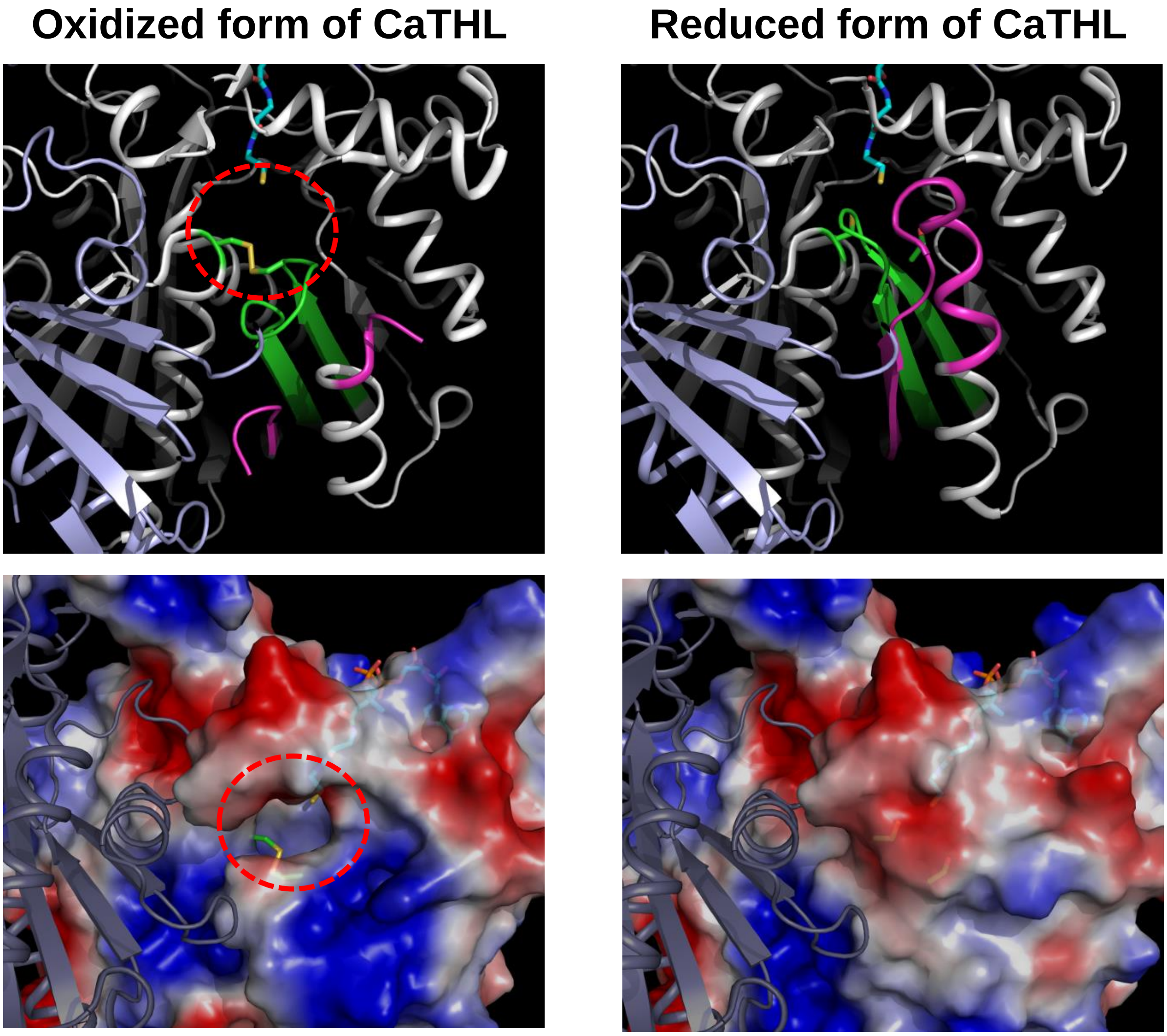

Supplementary Figure 12. Hole that chemical reductants can access to the disulfide bond in the oxidized form of *Ca*THL. (Top figures) CaTHL structures were shown as cartoon diagram. Two polypeptides were distinguished with gray and light blue colors. The disulfide bond and the “catalytic cysteine loop” were shown as green color and the “RDR” was as magentas color. (Bottom figures) One polypeptide was shown as electrostatic potential surface model and the other as a cartoon diagram. A hole that was found in the oxidized form of *Ca*THL was indicated by red colored dotted-circles.

Supplementary Table 1. The five χ angles of the disulfide bonds of *Ca*THL and *Ak*KAT.

**Protein**	**PDB ID**	**Chain/Residue**	**χ1**	**χ2**	**χ3**	**χ2'**	**χ1'**	**Bond designation**
*Ca*THL	4XL2	A (Cys88-Cys378)	169.84	-89.35	-102.04	-48.45	-43.52)	+/-LHSpiral
		B (Cys88-Cys378)	175.01	-93.47	-102.32	-51.02	-41.00	+/-LHSpiral
*Ak*KAT	2C7Y	A (Cys138-Cys192)	-61.73	-71.72	-86.84	-52.34	-67.44	-LHSpiral
		B (Cys138-Cys192)	-63.86	-67.38	-91.73	-53.74	-66.61	-LHSpiral

Supplementary Table 2. Primers used for cloning and site-directed mutagenesis of THLs.

Name	Primer
*Ca*THL_F	5′-TATA**CATATG**ATGAAAGAAGTTGTAATAGC -3′
*Ca*THL_R	5′-GGTG**CTCGAG**GCACTTTTCTAGCAATATTGC -3′
*Zr*THL_F	5′-CCC**CATATG**AGCACCCCGTCCATCGTC-3′
*Zr*THL_R	5′-CCC**AAGCTT**AAGGCTCTCGATGCACATCGC-3′
*Ec*THL_F	5′-CC**GGATCC**ATGAAAAATTGTGTCATCGTCAGTGC-3′
*Ec*THL_R	5′-CC**CTCGAG**TTAATTCAACCGTTCAATCACCATCG-3′
*Ca*THL^{C378S}_F	5′-GGCTTAGCAACTTTATCTATAGGTGGCGGAC-3′
*Ca*THL^{C378S}_R	5′-GTCCGCCACCTATAGATAAAGTTGCTAAGCC-3′
*Ca*THL^{V77Q}_F	5′-CTTTTAAAGCAGGATTACCACAAGAAATTCCAGCTATG-3′
*Ca*THL^{V77Q}_R	5′-CATAGCTGGAATTTCTTGTGGTAATCCTGCTTTAAAAG-3′
*Ca*THL^{N153Y}_F	5′-GATTGTGGGATGCATTTTATGATTACCACATGG-3′
*Ca*THL^{N153Y}_R	5′-CCATGTGGTAATCATAAAATGCATCCCACAATC-3′
*Ca*THL^{A286K}_F	5′-CAGGAGTTGACCCAAAAATAATGGGATATGG-3′
*Ca*THL^{A286K}_R	5′-CCATATCCCATTATTTTTGGGTCAACTCCTG-3′

Supplementary Table 3. Primers used for *in vivo* study of *Ca*THL.

Name	Primer
THL_F	5′-ATAT**CTGCAG**ATGAAAGAAGTTGTAATAGC-3′
THL_R	5′-ATAT**CCCGGG**ATTCAATTTACGCCTAGTAC-3′
THL/N153Y_F	5′-TATGATTACCACATGGGAAT-3′
THL/N153Y_R	5′-AAATGCATCCCACAATCCGT-3′
THL/A286K_F	5′-AAAATAATGGGATATGGACC-3′
THL/A286K_R	5′-TGGGTCAACTCCTGCTGAAC-3′
THL/V77Q_F	5′-GAAATTCCAGCTATGACTAT-3′
THL/V77Q_R	5′-TTGTGGTAATCCTGCTTTAAA-3′
